# Molecular Dynamics Simulations of a Chimeric Androgen Receptor Protein (SPARKI) Confirm the Importance of the Dimerization Domain on DNA Binding Specificity

**DOI:** 10.3389/fmolb.2020.00004

**Published:** 2020-01-31

**Authors:** Mahdi Bagherpoor Helabad, Senta Volkenandt, Petra Imhof

**Affiliations:** Department of Physics, Freie Universität Berlin, Berlin, Germany

**Keywords:** androgen receptor, glucocorticoid receptor, response element, protein-DNA interaction, chimeric SPARKI protein

## Abstract

The DNA binding domains of Androgen/Glucocorticoid receptors (AR/GR), members of class I steroid receptors, bind as a homo-dimer to a cis-regulatory element. These response elements are arranged as inverted repeat (IR) of hexamer “AGAACA”, separated with a 3 base pairs spacer. DNA binding domains of the Androgen receptor, AR-DBDs, in addition, selectively recognize a direct-like repeat (DR) arrangement of this hexamer. A chimeric AR protein, termed SPARKI, in which the second zinc-binding motif of AR is swapped with that of GR, however, fails to recognize DR-like elements. By molecular dynamic simulations, we identify how the DNA binding domains of the wild type AR/GR, and also the chimeric SPARKI model, distinctly interact with both IR and DR response elements. AR binds more strongly to DR than GR binds to IR elements. A SPARKI model built from the structure of the AR (SPARKI-AR) shows significantly fewer hydrogen bond interactions in complex with a DR sequence than with an IR sequence. Moreover, a SPARKI model based on the structure of the GR (SPARKI-GR) shows a considerable distortion in its dimerization domain when complexed to a DR-DNA whereas it remains in a stable conformation in a complex with an IR-DNA. The diminished interaction of SPARKI-AR with and the instability of SPARKI-GR on DR response elements agree with SPARKI's lack of affinity for these sequences. The more GR-like binding specificity of the chimeric SPARKI protein is further emphasized by both SPARKI models binding even more strongly to IR elements than observed for the DNA binding domain of the GR.

## 1. Introduction

Steroid receptors (SRs), a subfamily of nuclear receptors, are ligand-activated transcription factors that bind to a specific DNA target sequence in order to enhance or repress gene transcription (Evans, 1988; Corson, 2005; Bunce and Campbell, 2010).

**Figure 1:**
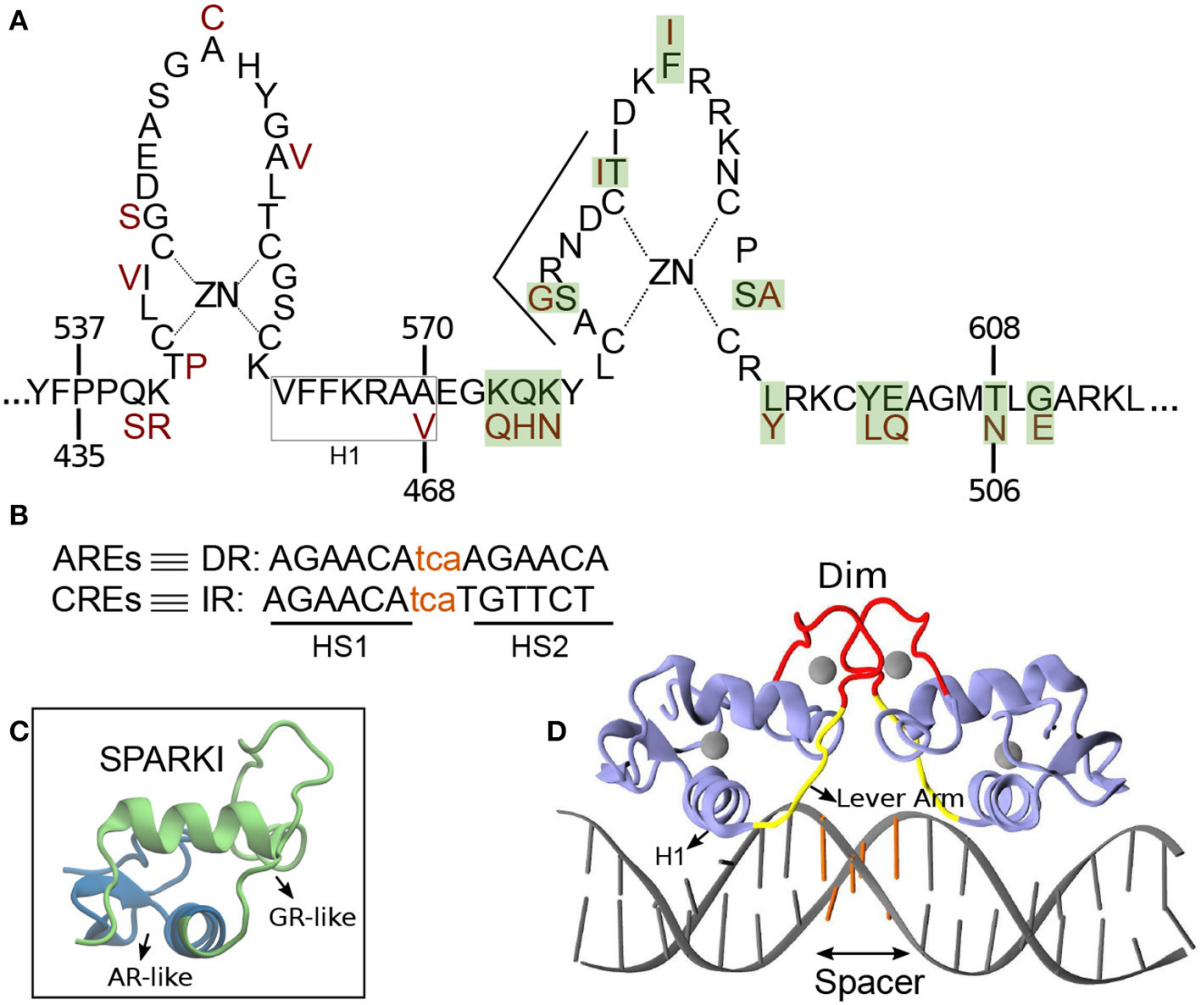
**(A)** Schematic overview of the DNA binding domain (DBD) sequences in the androgen receptor (AR) and glucocorticoid receptor (GR) protein with corresponding residue numbers above and below, respectively. The amino acids colored in dark red are those elements of the GR-DBD that differ from the AR-DBD sequence. The other amino acids are the same in the AR- and GR-DBD. The amino acids shown with green shadow are those elements in AR that are replaced with residues from GR in order to make Sparki (Schauwaers et al., 2007). **(B)** DNA sequences for direct (DR) and inverted repeats (IR). The non-capital letters are the spacer base-pairs, colored in orange. **(C)** Schematic 3D structure of one monomer of Sparki-DBD, regions colored in green and blue are those subdomains that are GR- and AR-like, respectively. **(D)** The 3D structure of the GR- DBD/DNA complex (pdb ID: 1R4R). A similar structure exists for the AR-DBD/DNA complex (pdb ID: 1R4I). The lever arm and dimerization domain (Dim) are shown in yellow and red, respectively. The spacer region of the DNA is colored with orange.

Members of SRs, i.e., Androgen receptor (AR), Glucocorticoid receptor (GR), Mineralocorticoid receptor (MR), and Progesterone receptor (PR), bind as a homo-dimer to consensus 15 base pair (bp) palindromic DNA sequences, termed classical response elements (CREs) (Ham et al., 1988). The DNA of CREs is organized as an inverted repeat (IR) of hexamer “AGAACA”, separated with a 3 bp DNA sequence, called spacer (Beato et al., 1995) (Figures 1B,D). Among the CREs, the first hexamer (HS1) elements are almost invariant and therefore suggested as high affinity DNA sequences for receptor binding (La Baer and Yamamoto, 1994). The DNA binding domain (DBD) of the proteins, which includes about 70 amino acid residues, contains two vital subdomains, each identified with a zinc ion that is coordinated by four Cysteine residues. The first subdomain includes an α-helix, termed H1, which is responsible for protein-DNA major groove interactions. The second subdomain holds a loop domain, termed Dim, which is responsible for protein-protein dimerization (Luisi et al., 1991; Kumar and Thompson, 1999) (see Figures 1A,D). A flexible loop, named lever arm connects these subdomains to each other (Figure 1D).

Steroid receptors show high structural conservation and share almost identical DNA response elements, allowing these response elements to be functionally substituted (Arora et al., 2013). For instance, a response element that corresponds to the androgen receptor might function for glucocorticoid receptor activation and vice versa. Recent studies have shown that AR and GR share about one third of their response binding sites (Zhang et al., 2018). Still, androgen response elements (AREs) are merely recognized by AR and not by GR (Schoenmakers et al., 1999; Claessens et al., 2001; Moehren et al., 2008). The AREs are arranged as direct-like repeat (DR) “TGTTCT” of hexamer “AGAACA” (see Figure 1B) and also separated with a 3 bp spacer (Haelens et al., 2003). In 2004, Shaffer et al. crystallized the only structure of AR(DBD) in complex with a DR response element in which an unexpected head-to-head conformation was revealed (Shaffer et al., 2004). This structure of AR-DR indicates additional hydrogen-bond interactions of residue S580, which is not present in GR, in each monomer with its counterpart in the other monomer. These interactions have been discussed as a potential stabilization of the unexpected head-to-head arrangement in the AR-DR complex (Verrijdt et al., 2003; Shaffer et al., 2004).

Studies have shown that AR activity varies depending on the bound response elements, i.e., DR or IR (Geserick et al., 2003; Verrijdt et al., 2006). For instance, R581D mutation in the dimerization domain of AR-DBD enhances AR's activity on CREs but has less effect on AREs. On the other hand, the A579T mutation shows reduced activity on AREs but not on CREs (Geserick et al., 2003). In contrast, mutations at points that differ between the AR and GR Dim, i.e., S580G and T585I, in the AR, and G478S and I483T, in the GR, do not show much effect on DNA binding affinity and activity of these receptors (Verrijdt et al., 2006). These mutation data indicate that less of the AR-DR binding specificity can be attributed to the Dim interface than suggested by the crystal structure. Also, it is shown that the changes in AR activity due to the loss of Dim interactions strongly depend on the engaged DNA response element (van Royen et al., 2012). Since the Dim region is too far (about 18 Å) from the DNA surface to build direct interaction, other parts of DBDs likely play a role in DNA binding specificity (Meijsing et al., 2009). In a recent study, Watson et al. showed that the lever arm conformation strictly depends on the spacer sequence. The lever arm has therefore been suggested as an allosteric modulator that not only connects the H1 to the Dim (see Figure 1), but also associates the DNA response sequence to its respective dimer partner (Watson et al., 2013). The activities of AR and GR are shown to also depend on this region (Meijsing et al., 2009; Helsen et al., 2012; Dalal et al., 2014). A recent study on the DNA-binding preferences of AR and GR has revealed that AR binding to DNA is more enthalpically energized, while GR binding is more entropy driven (Zhang et al., 2018).

In 2007, an *in vivo* study done by Schauwaers et al. generated a chimeric receptor, termed SPARKI (SPecificity-affecting AR KnockIn), in which 12 amino acids of AR in its second zinc-binding domain were replaced by those of GR (Figures 1A,C) (Schauwaers et al., 2007). *In vitro* studies have shown that swapping this second zinc-binding motif between the AR and GR leads to the loss of affinity of this chimeric receptor with a DR-like motif (Schoenmakers et al., 1999; Moehren et al., 2008). Consistently, the *in vivo* experiment exhibited a reduced affinity of the SPARKI receptor for DR-like elements whereas for IR-like elements it showed similar or even better binding affinity than AR (Schauwaers et al., 2007). The lack of the SPARKI system's ability to bind to DR-like response elements was also confirmed by a later *in vivo* study, done by Sahu et al. (2014). Interestingly, this study shows that for DR-like elements, which were selectively enriched by wild-type AR, there is a well-conserved 5′-hexamer (HS1, Figure 1B) but not a stringent 3′-hexamer (HS2) sequence conservation. In contrast, binding of both wild-type AR and SPARKI to IR-like elements requires a specific HS2 sequence (Sahu et al., 2014). Moreover, *in vitro* assays show the high-affinity of AR and GR receptors to HS1, due to its highly conserved sequences (Verrijdt et al., 2000). It is speculated that due to the high-affinity of the two subunits in the AR dimer, this receptor could bind to a more diverse HS2 than the GR could. For instance, it is shown that the thymine (T) next to guanine (G) in HS2 of the IR elements is a highly conserved base in the response elements of SRs. This specific T is not required for AR, allowing this receptor to bind to DR-like elements which have an adenine (A) in that position (John et al., 2011; Sahu et al., 2011, 2014; Yin et al., 2012; Ballaré et al., 2013; Grøntved et al., 2013). However, it is not yet clear how the high affinity of AR-DBD to DR-like response elements, which leads to strong interactions in the protein's dimerization interface, is influenced by (more diverse) HS2 elements. Moreover, the distinct binding of AR(DBD)-DR (or IR) and GR(DBD)-IR is still not well-understood. The SPARKI is an outstanding model that could explain the distinct regulation of AR-specific responses with respect to those which can be regulated by GR as well.

In this study, by employing all-atom molecular dynamics simulations, we investigate the factors that lead to a different binding of AR and GR receptors to DNA response elements. In this regard, we simulated six protein-DNA complexes consisting of the DNA binding domains of wild type AR and GR, bound to a DNA sequence with IR and DR, respectively, and SPARKI models (with both IR and DR elements) made by AR and GR mutation. Our MD simulations allowed us to determine the significant dynamics of these receptor's DBD-DNA interface. These results suggest a loss of affinity of the chimeric proteins, i.e., SPARKI, to DR sequences and a strong affinity for IR sequences. Furthermore, our data reveal that the “weaker” dimerization interface interactions in the IR complexes, compared to the AR-DR complex, allows those dimeric proteins to be properly accommodated on IR sequences.

## 2. Materials and Methods

### 2.1. Structural Models

The atomic models of the DNA binding domains (DBD) of AR- and GR complexed to their respective response element were prepared using the crystallographic structures 1R4I and 1R4R, respectively. In order to achieve consistency with the AR(DBD)-DNA complex, the guanine in the spacer region of the GR(DBD)-DNA complex, was mutated *in silico* to cytosine. The response elements in the two complexes are thus 5′-CC **AGAACA**tca**TGTTCT** GA-3′ (DR, for AR) and 5′-CC **AGAACA**tca**AGAACA** GA-3′ (IR, for GR), respectively. The residues listed in bold are the core response elements including the two half sites, HS1 and HS2, respectively, the spacer is given in small letters. We have constructed two atomic models of the SPARKI receptor, one based on the structure of the AR-DNA complex (1R4I) and one on the structure of the GR-DNA complex (1R4R). In the AR-based model, termed SpAR, residues in the second zinc-binding motif of AR that differ from GR (highlighted in green in Figure 1A), were replaced with the corresponding residues of the GR protein, as in the experimental mutation (Schauwaers et al., 2007). These residues are located at the dimerization interface (see Figures 1A,C). The second model, termed SpGR, is based on the GR protein in which the residues of the first zinc-binding motif of GR that differ from AR, which are part of the DNA-binding interface, were mutated to those of AR. The resulting sequence of the proteins in both Sparki models, SpAR and SpGR is thus identical, however, their initial structures differ, since these are based on two different crystal structures.

Both SPARKI models were furthermore modeled in complex with both DNA sequences, DR and IR, respectively. Therefore, a total of six models, i.e., AR-DR, GR-IR, SpAR-DR, SpAR-IR, SpGR-DR, and SpGR-IR have been simulated.

### 2.2. Molecular Dynamics Simulations

The systems were solvated with ~23,000 water molecules in a cubic box of ~90 × 90 × 90 Å^3^ and a number of sodium ions were added to neutralize the systems. The CHARMM-27 force field (Brooks et al., 1983; MacKerell et al., 1998) and the TIP3 water model were used in the simulations (Mahoney and Jorgensen, 2000). Long-range electrostatic interactions were treated by the particle mesh Ewald method via a switch function with a cutoff of 14–12 and employing periodic boundary conditions (Darden et al., 1993). The systems were energy minimized for 5,000 steps (conjugate gradient with an energy tolerance of 10^−4^ kcal/mol), followed by a molecular dynamics (MD) simulation of 30 ps (time step of 1 fs) to heat the system by velocity scaling (with harmonic constraint on all heavy atoms, by force constant 10 kcal·mol^−1^·^−2^). Then, 100 ps of MD relaxation (in NPT ensemble) at target temperature (300 K) and time step 1 fs were computed. Langevin dynamics with a damping factor of 1 ps^−1^ have been used for temperature control (Allen and Tildesley, 2017). The Nosé–Hoover Langevin pressure control, with piston period of 200 fs and a damping time of 100 fs, have been used in order to maintain the pressure at 1 bar (Martyna et al., 1994). After the equilibration phase, three 100 ns MD replicas (with different initial velocities) for each system were carried out (time step of 2 fs). From those, one run per system was chosen for longer simulation, based on the calculated root mean-squared deviation (RMSD) (see Figure S1). These longer MD simulations were carried out for 900 ns for the SPARKI systems and for 500 ns for AR-DR and GR-IR, respectively, and saved at 2 ps intervals. In all simulations, the terminal DNA base pairs were restrained (centered around 3 Å between the centers of mass of the respective bases) by a harmonic potential with a force constant of 20 kcal/mol in order to decrease the edge effects. The MD simulations were run using version 2.10 of NAMD (Phillips et al., 2005).

### 2.3. Hydrogen Bond Analysis

Hydrogen bonds were analyzed based on geometric criteria, i.e., a maximal distance of 3.2 Å between donor and acceptor atom and an angle formed by donor, hydrogen atom, and acceptor, that deviates maximally by 42° from linear. This criterion was evaluated for each frame of the simulation trajectory, i.e., each 2ps of the simulations time. A hydrogen-bond probability is then obtained as the hydrogen bond occupancy $H_{occ}=\frac{n_{Hbond}}{N}$, i.e., the number of frames in which a hydrogen bond is formed, *n*_*Hbond*_, divided by the number of frames analyzed, *N*. Water-mediated hydrogen-bonds between protein and DNA were identified as two hydrogen bonds formed simultaneously by a water molecule, one with the protein and another one with the DNA. The hydrogen bond analysis has been carried out using VMD (Humphrey et al., 1996) and in-house scripts.

### 2.4. Conformational Analysis

The median structure of each trajectory was determined as the snapshot that has minimum root mean-squared deviation (RMSD) from the averaged structure of the trajectory. The local DNA conformation was analyzed using Curves+, a program for analyzing the coarse-grained geometry of DNA (Lavery et al., 2009). The errors estimated for the DNA parameters are standard errors, which are calculated by a block averaging approach (Grossfield and Zuckerman, 2009).

### 2.5. Linear Correlation Score Function

Correlations between all pairs of fluctuating atom positions were calculated as Pearson correlation. The Pearson correlation, is defined by the normalized covariance matrix (Ichiye and Karplus, 1991):

(1) $$\begin{array}{r} r_{ki}=\frac{cov(x_{k},x_{i})}{\sigma_{x_{k}}\sigma_{x_{i}}} \end{array}$$

where ***x***_***k***_ and ***x***_***i***_ are the fluctuations of random variable *k* and *i*, respectively.

The correlation score function is a measure of the intensity of correlation for each variable *k* (here, the position of the Cα atoms of the protein residues), defined as (Ricci et al., 2016):

(2) $$\begin{array}{r} CS_{k}=\frac{1}{N-1}\overset{N-1}{\underset{i}{\sum }}r_{ki} \end{array}$$

Here, the correlation score function is normalized. In order to remove the trivial and non-important correlations only pairs with a of *r*_*ki*_ ≥ 0.4 were considered.

### 2.6. Entropy Estimation

The configurational entropy of the protein is estimated based on the mass weighted covariance matrix of atomic fluctuations via two well-established methods, one proposed by Schlitter (Schlitter, 1993) and another one by Andricioaei and Karplus (2001).

For computation of the protein entropy we used the fluctuations of the backbone Cα atoms. The last 300 ns of the simulations are considered for the analysis. The error bars are standard deviation of three different simulation trajectories samples due to different chosen time strides. All the calculations are done via Grcarma software, a Task-Oriented Interface for the Analysis of MD trajectories (Koukos and Glykos, 2013).

## 3. Results

The results are organized to first present a comparison of the overall structure of the complexes. This is followed by an analysis of the proteins, first, in terms of flexibility and an estimate of their entropies in the different complexes. Then, the protein-protein interactions between the two subdomains are investigated. Subsequently, the conformation of the two DNA sequences in the different complexes is analyzed. Finally, the hydrogen-bond interactions between the proteins and the DNA are reported.

### 3.1. Median Structure

**Figure 2:**
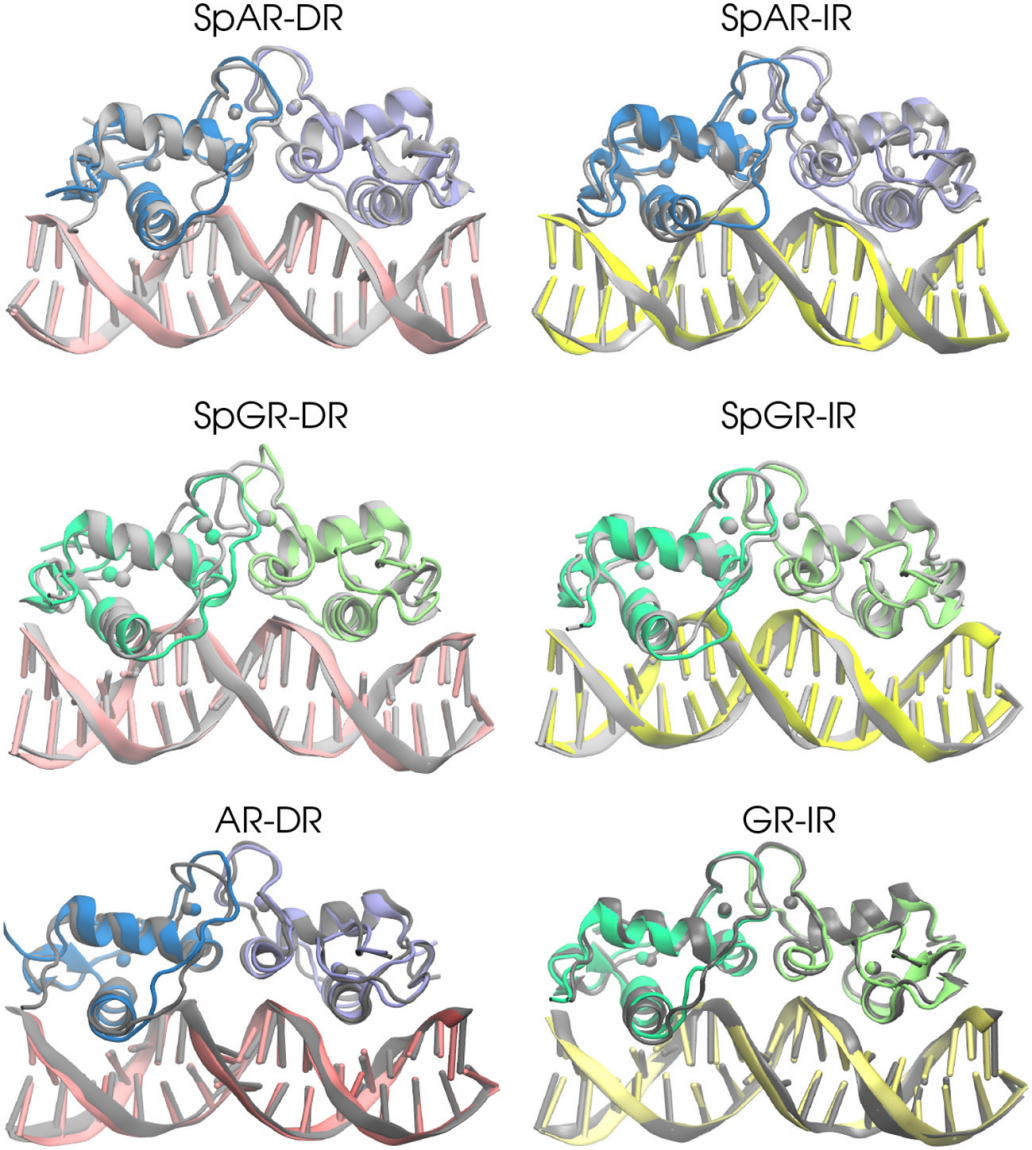
The 3D median structures of the complexes. In each system, the median structure of the last 100 ns of simulation (colored) is aligned to the median structure of the first 100 ns simulation (gray).

In order to estimate the overall structural change of each complex during the simulation, the median structures representing the first 100 ns and last 100 ns (of the total of 500 ns simulation time for AR-DR and GR-IR, respectively, and 900 ns for SPARKI models), respectively, were aligned with respect to each other and compared. As can be seen in Figure 2, the lever arm is the most variable domain whereas the initial and final conformations of the remainder of the systems are similar. Remarkable exceptions are the monomer A, located at the first half-site, and the Dim interface of the SpGR-DR model, which exhibit a considerable distortion. In this model, a conformational change takes place not only in the lever arm but also in both zinc-binding subdomains where the zinc ions, together with their coordinating ligands, change positions. Moreover, the Dim regions of the AR-DR system are slightly closer to each other than in the other models. The distances between different domains/subdomains of protein-DNA complexes are listed in Table S1. As shown in this table, the distance between monomer A and monomer B in AR-DR (24.37 ± 0.31 Å) is shorter than that of GR-IR (25.08 ± 0.20 Å). The SpGR-DR system also exhibits a larger distance between the receptor's dimer interfaces as well as between the respective zinc ions of the two subunits, than the other systems. The simulations of the SpAR-DR model, which represent the same system but were started from a different initial structure, in contrast, do not exhibit a distortion of the Dim interface, Accordingly, the distance between the two monomeric subunits in this model are shorter than in the SpGR-DR model.

### 3.2. Root Mean Square Fluctuations (RMSF)

**Figure 3:**
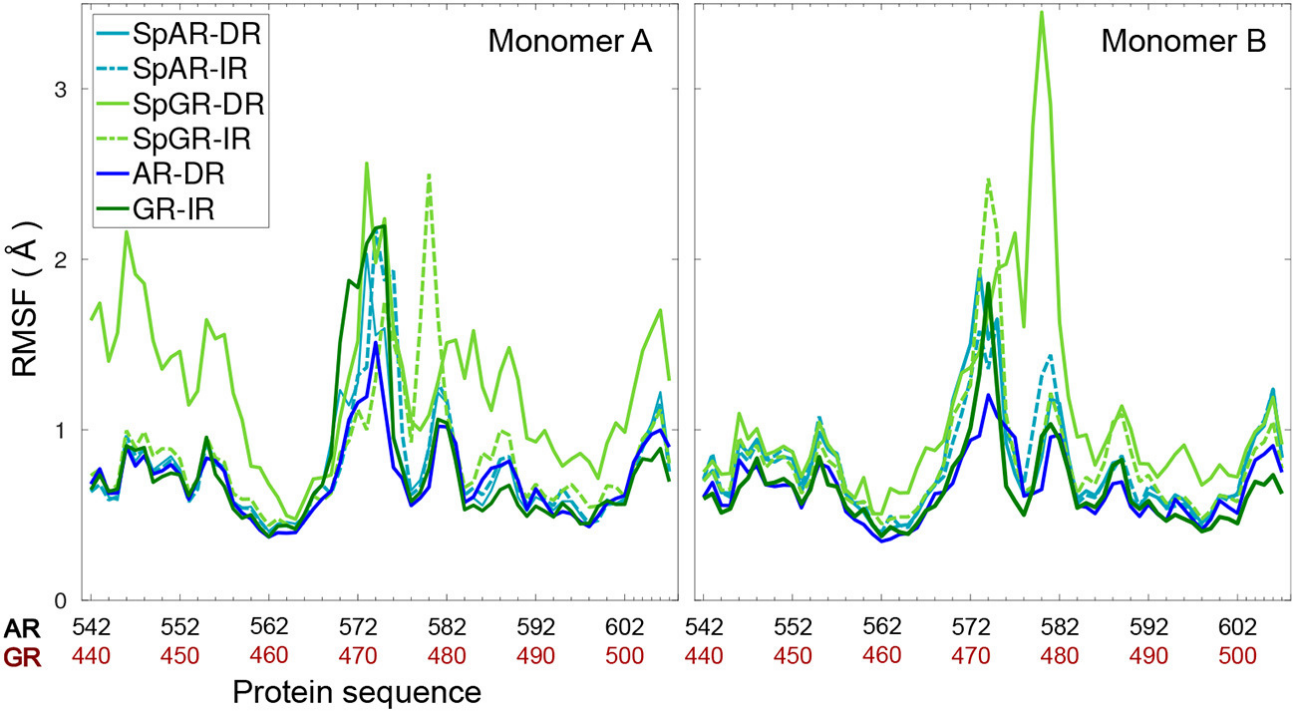
Per-residue root mean square fluctuations of Cα atoms of the protein (monomer A&B) for all systems.

Figure 3 shows the per-residue root mean square fluctuations (RMSF) of the protein monomers for all the systems. As can be seen in this figure, the lever arm corresponding to residues 571–576 (AR, SpAR)/469–474 (GR, SpGR) is the most fluctuating region in all models. Comparison of fluctuations between monomer A and monomer B shows almost similar fluctuations of the protein residues in all systems, except for SpGR-DR. The IR complexes, though, exhibit higher flexibility than the DR complexes in the lever arm region, i.e., residues 469–474 or 571–576 in GR or AR numbering, respectively. SpGR-DR exhibits particularly high fluctuations of the protein residues, especially in monomer A; higher than the fluctuations of monomer A in any of the other systems. Monomer B of SpGR-DR, however, shows larger fluctuations than the other systems only for the residues situated in the dimer interface, i.e., 576–581 (AR, SpAR)/474–479 (GR, SpGR). Of note, in the SpGR models, residues in the dimer interface are directly modeled, that is without *in silico* mutation, from the crystal structure of the wild-type GR protein and may therefore represent a GR-like conformation.

### 3.3. Entropy Estimation

**Figure 4:**
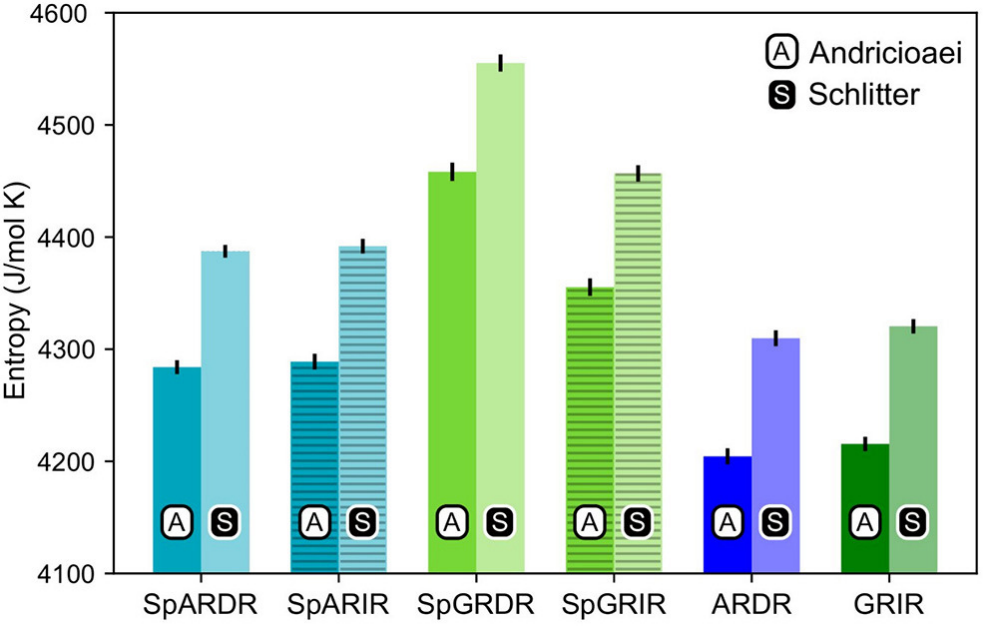
Entropy estimates for the proteins of all complexes. The first and second columns, shown with black **A** and white **S** are the entropy values estimated with the Andricioaei and Schlitter models, respectively.

As can be seen from Figure 4, the estimated entropy of SpGR-DR and SpGR-IR are higher than those computed for SpAR-DR and SpAR-IR, respectively. This is the case for both entropy estimation methods. Both AR-DR and GR-IR exhibit rather similar values in entropy, although the two proteins are in complex with different DNA sequences. Comparison of only DR or IR complexes, respectively, shows higher entropy values for the Sparki models than for the respective wild-type complexes. Among the chimeric Sparki models, SpAR does not exhibit a significant difference in entropy when complexed to DR or IR sequence, whereas SpGR shows a significantly higher entropy in the DR complex compared to the IR complex.

### 3.4. Protein–Protein Hydrogen Bond Interactions

The hydrogen bond interactions between the protein subunits are listed in Table 1. Our results indicate that the dimer interface of the AR-DR system forms more strong hydrogen-bond interactions than those seen in the SPARKI systems and in the GR-IR. In particular, the inter-subunit hydrogen bond S580_*A*_-S580_*B*_, which has been discussed to be crucial for tight dimerization of the AR-DR complex (Shaffer et al., 2004), is not present in the other systems. Furthermore, a strong interaction of R581-D583 can also be seen in AR-DR, but not in the other systems. Two interactions, L577-N593(AR, SpAR)/L475-N491(GR, SpGR) and A579-I585(AR, SpAR)/A477-I483(GR, SpGR), exist in all the systems, in both directions, that is from monomer A to monomer B (AB) and vice versa (BA). However, in the SpGR-DR, only a one-sided of these interactions is formed, indicating a weaker dimer interface interaction of the SpGR-DR than in the other systems. Moreover, the dimer interfaces of the SpAR complexes exhibit stronger hydrogen-bond interactions than the SpGR models. An extra interaction of C578-R590 can be seen in SpARs that is not present in SpGRs. This extra interaction is also observed in the AR-DR complex, based upon which the SpAR-DR model has been built. The dimerization interactions of the GR-IR model also exhibit two moderate and one-way (BA-side) hydrogen-bond interactions C476-R488 and R479-D481 that are not present in SpGR models.

**Table 1:** Protein-protein hydrogen-bond interactions.

	**AR-DR**		**SpAR-DR**		**SpAR-IR**	
	**AB**	**BA**	**AB**	**BA**	**AB**	**BA**
L577-N593	62%	44%	72%	70%	51%	55%
A579-I585	89%	93%	95%	91%	95%	88%
C578-R590	–	47%	60%	–	71%	52%
R581-D583	100%^*^	100%^*^	–	–	–	–
S580-S580	80%	80%	–	–	–	–
S580-D583	–	48%	–	–	–	–
	**GR-IR**		**SpGR-DR**		**SpGR-IR**	
	**AB**	**BA**	**AB**	**BA**	**AB**	**BA**
L475-N491	59%	74%	–	90%	71%	51%
A477-I483	91%	85%	81%	–	92%	66%
C476-R488	–	66%	–	–	–	–
R479-D481	–	59%^*^	–	–	–	–

*The star indicates that more than one hydrogen bond is formed simultaneously. AB and BA refer to the monomer A as donor and monomer B as acceptor and vice versa. Here, the hydrogen-bond interaction occupancies below 40% are considered as weak interactions and are therefore not listed*.

### 3.5. Linear Correlation Score

**Figure 5:**
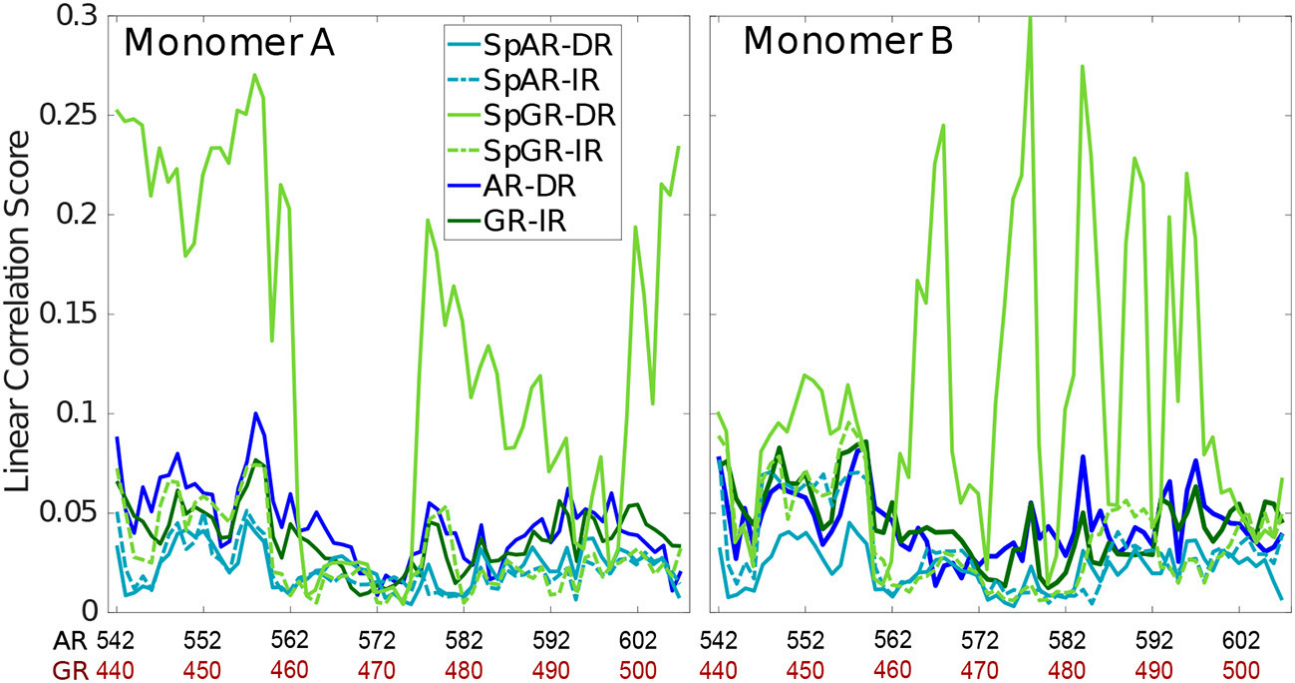
Correlation score per residue, computed for intra-domain correlations with *r*_*ki*_ ≥ 0.4.

In order to capture how the protein residues in each monomer are influenced by other residues of that monomer, the linear correlation score has been calculated for all the systems (linear correlation scores calculated for the first 100 ns and middle 100 ns of trajectories of the SPARKI systems are shown as Supplementary Material, see Figure S13). As can be seen in Figure 5, almost all the residues show a similar magnitude of correlation score in all the systems, except for SpGR-DR. This model exhibits considerably higher correlation score values, in both protein monomers, than any of the other models. This indicates that the fluctuating motion of each residue is highly dependent on the rest of the residues in that protein. Any local conformational change, as observed for the lever arm and the Dim of SpGR-DR, as visualized by the median structures (see above), does not only affect the neighboring residues but also distal domains of the protein and thus has a more global effect. Moreover, for SpGR-DR the correlation score increases during the simulation, corresponding to an increase in conformational change of the monomers in this model (see Supplementary Figure S13).

### 3.6. DNA Conformation

**Figure 6:**
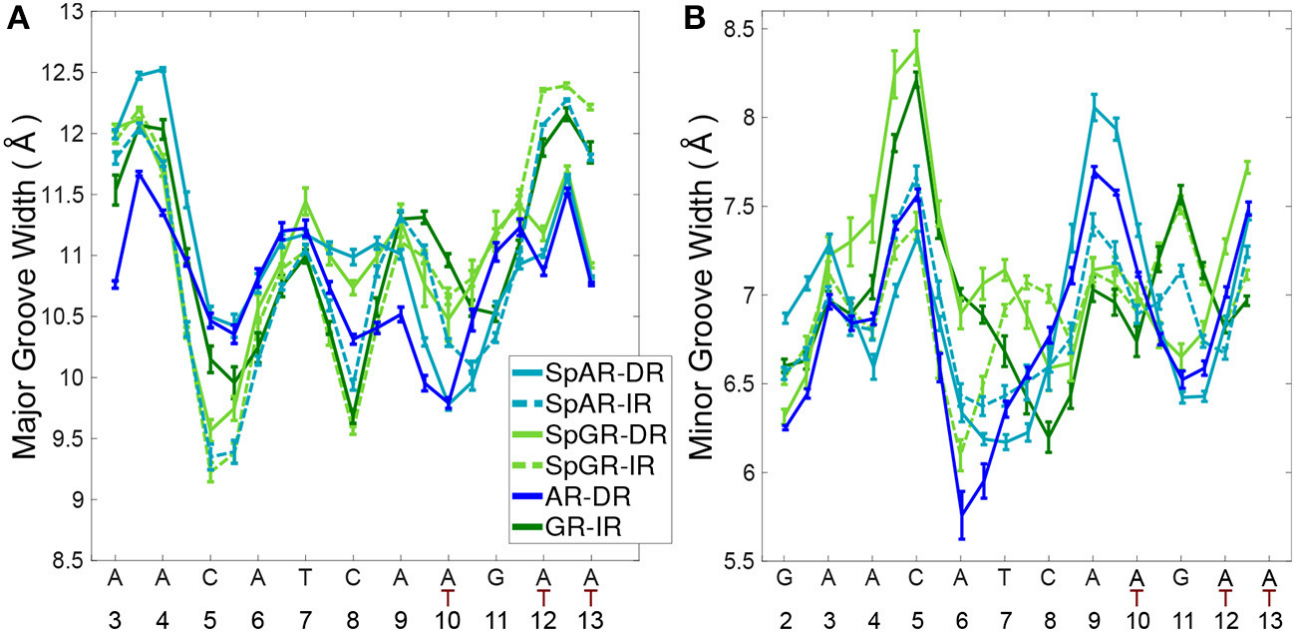
The DNA **(A)** major groove and **(B)** minor groove widths for all systems.

To study the impact of the DBD of the receptors on their respective DNA structure, the local geometrical parameters of DNA, i.e., inter- and intra-bp parameters (Figures S5–S10), major- and minor-groove widths (Figure 6), and helical axis bending (Figure S4) were calculated for the last 100 ns of the AR-DR and GR-IR trajectories. For the SPARKI systems, the changes of these parameters in the course of the simulations were also considered (Figures S2, S3) and are discussed in the Supplementary Material.

The DNA grooves of the IR complexes differ from those of DRs. Interestingly, these differences can not only be observed in the second hexamer, which is expected due to the different DNA sequence, but also in the spacer and in the first hexamer in the IR complexes (see Figure 6). For instance, the major groove at position C8, in the spacer region, is narrower in the IRs than in DRs. Also, a narrower major groove at positions C5-A6 (in HS1) can be observed in Sp(AR/GR)-IR compared to SpAR-DR or AR-DR. The DNA of both SPARKI-IR systems exhibits very similar conformations. This can be seen in almost all DNA parameters (see Figures S5–S10).

The DNA parameters in both SPARKI-IR complexes show some differences from the GR-IR parameters. The minor groove of Sp(AR/GR)-IR at positions between A4-T7 (in HS1) is narrower than that in the GR-IR (see Figure 6). Also, the DNA of the GR-IR complex shows higher bending than the DNA of the Sp(AR/GR)-IR complexes (Figures S4B,D). Since the DNA sequence is the same in all IR complexes, the observed differences in the DNA conformation can be attributed to the interaction with the different proteins.

In contrast to the two SPARKI-IR complexes, all DNA parameters of the SpAR-DR complex and the SpGR-DR complex represent conformations that are considerably different from AR-DR (see Figure 6 and Figures S4–S10). SpAR-DR and SpGR-DR, moreover, show differences between some of their DNA parameters. For instance, in SpGR-DR the HS2 has a wider major and narrower minor groove and HS1 has a considerably wider minor groove than in SpAR-DR. Furthermore, the DNA helical axis bending is higher in SpGR-DR than in SpAR-DR (Figure S4). In the two SPARKI-DR models not only the DNA sequence is the same, but also the residues of the protein. The different DNA conformations may also be attributed to different interactions with the (same) proteins, representing different (metastable) binding modes due to different initial starting conformations.

In the SPARKI-IR systems, the first hexamer exhibits a narrower major groove than the second hexamer whereas the opposite is observed for the SpAR-DR and AR-DR systems (see Figure 6). Interestingly, the position T_12_, in the second hexamer, seems to have an important role in the IR complexes. For most IR complexes the dinucleotide G_11_T_12_ shows an extreme value which is not the case in the DNA parameters of the DRs with G_11_A_12_ at this position (see Figures S5, S6, S9). Also the intra base pair parameters exhibit at position G_11_ more extreme values in the IR complexes than in those with DR (Figures S7, S8, S10), which may be an effect of the neighboring residue being thymines at positions T_10_ and T_12_ in IRs, instead of adenine residues in DRs.

### 3.7. Protein-DNA Hydrogen-Bond Interactions

**Figure 7:**
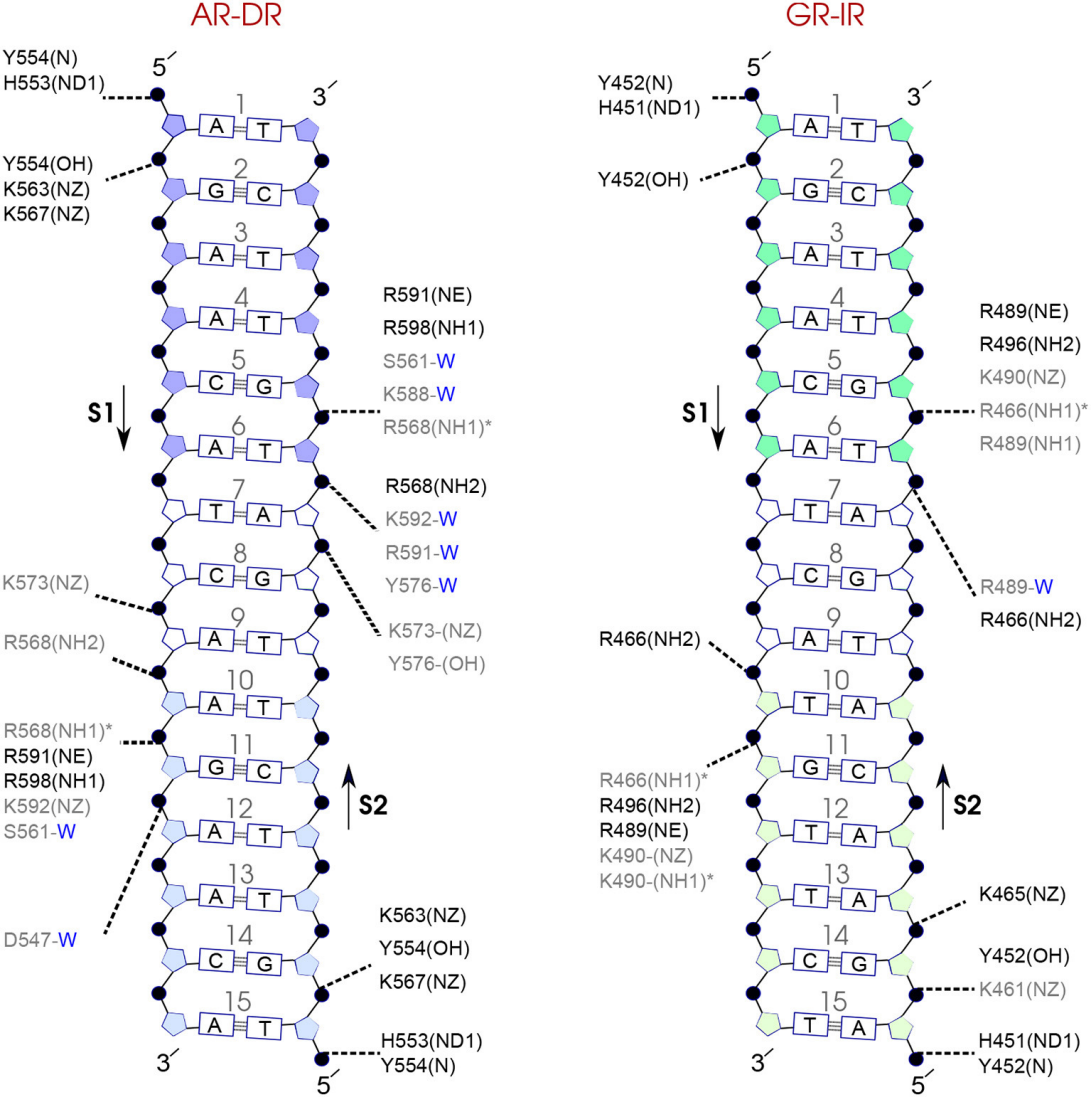
Diagram of protein-DNA hydrogen-bond interactions for **(left)** AR-DR and **(right)** GR-IR. The nucleotides of the 15 bps core DNA sequence are numbered from HS1 (numbers: 1–6) to HS2 (numbers: 10–15). The spacer region is highlighted with non-colored boxes around the numbers of the bases (numbers: 7–9). The hydrogen bonds are categorized based on their occupancy, 50–75% (gray), and 75–100% (black). The water mediated hydrogen bonds are shown with a blue letter “W.” The residues shown with star sign form base-specific hydrogen-bond interactions while the other residues interact with the backbone of the DNA.

**Figure 8:**
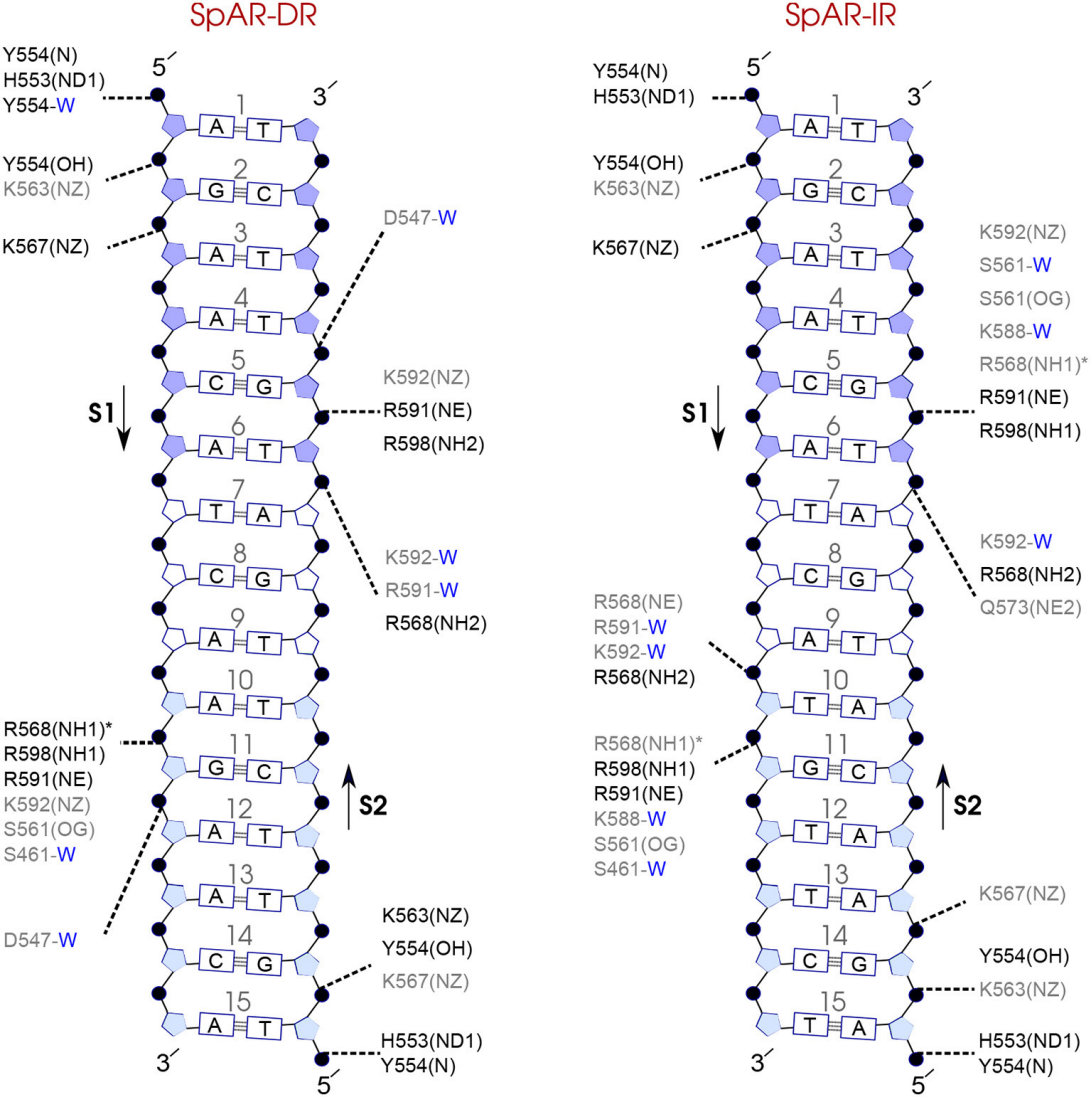
Diagram of protein-DNA hydrogen-bond interactions for **(left)** SpAR-DR and **(right)** SpAR-IR. The nucleotides of the 15 bps core DNA sequence are numbered from HS1 (numbers: 1–6) to HS2 (numbers: 10–15). The spacer region is highlighted with non-colored boxes around the numbers of the bases (numbers: 7–9). The hydrogen bonds are categorized based on their occupancy, 50–75% (gray), and 75–100% (black). The water mediated hydrogen bonds are shown with a blue letter “W.” The residues shown with star sign form base-specific hydrogen-bond interactions while the other residues interact with the backbone of the DNA.

**Figure 9:**
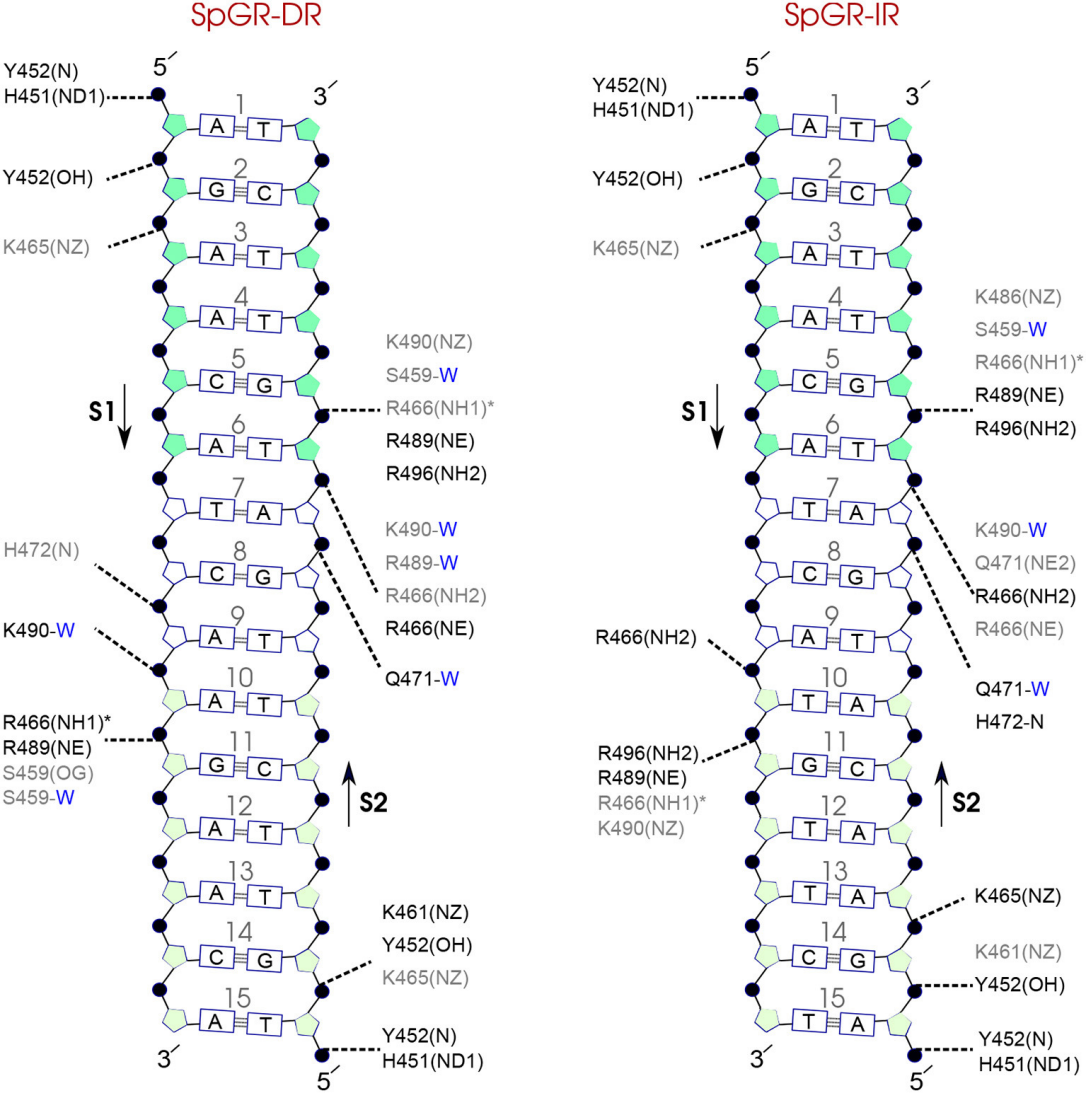
Diagram of protein-DNA hydrogen-bond interactions for **(left)** SpGR-DR and **(right)** SpGR-IR. The nucleotides of the 15 bps core DNA sequence are numbered from HS1 (numbers: 1–6) to HS2 (numbers: 10–15). The spacer region is highlighted with non-colored boxes around the numbers of the bases (numbers: 7–9). The hydrogen bonds are categorized based on their occupancy, 50–75% (gray), and 75–100% (black). The water mediated hydrogen bonds are shown with a blue letter “W.” The residues shown with star sign form base-specific hydrogen-bond interactions while the other residues interact with the backbone of the DNA.

In order to analyze the interaction strengths, probabilities of direct and indirect (mediated by water molecules) hydrogen bonds between protein and DNA have been calculated. Figures 7–**9** show the hydrogen bond interactions of all studied systems, calculated from the last 100 ns of the simulations. For the SPARKI systems, the hydrogen bond interactions of the middle 100 ns (W2 interval) were also calculated (see Figures S11, S12). According to these figures, differences in protein-DNA interactions between W2 and W3 intervals in SpARs can be seen only in the first hexamer, HS1 (Figure S11), whereas for SpGRs such differences exist in both DNA hexamers (Figure S12).

For each DNA hexamer, i.e., HS1 and HS2, there are four sites whose hydrogen bond interactions with the protein are conserved among all the systems. These are s1A1, s1G2, s2G5, and s2T6 in HS1 and s1A10, s1G11, s2T15, and s2G14 in HS2. The guanine residues at positions s1G11 and s2G5 are the predominant residues that form strong, i.e., highly probable, hydrogen-bond interactions with the protein in all systems. In particular, the residue R568 in the helix H1 of the AR-DBD, and residues R466 in helix H1 of the GR-DBD form base-specific hydrogen bonds with guanine residues s1G11 and s2G5, respectively. Our results indicate that the AR-DR complex involves more hydrogen-bonded protein-DNA interactions than the GR-IR complex. Moreover, hydrogen bonds of residues s1G2 and s2G14 with K563 and K567, respectively, and also those of residues s2A7 and s2T6 (in the spacer) with Y576 are stronger in the AR-DR complex than the corresponding hydrogen bonds in the GR-IR complex (see Figure 7).

Comparison of the hydrogen-bond patterns between the SpAR systems shows that the SpAR-IR complex has more strong and moderate hydrogen-bond interactions than the SpAR-DR complex. In particular, residues s1T10 and s2G5 are more strongly hydrogen-bonded in the SpAR-IR model than in the SpAR-DR complex (see Figure 8). The two SpGR systems show rather similar protein-DNA hydrogen-bond interactions (see Figure 9). However, comparing the hydrogen-bond interactions between the SpAR-IR and SpGR-IR shows that the SpAR-IR includes more and stronger hydrogen interactions than the SpGR-IR. In particular, for the SpAR-IR model more hydrogen bonds than in the SpGR-IR complex can be observed for each specific guanine residue, i.e., s1G11 and s2G5. One further residue, i.e., s1T10, forms stronger hydrogen-bonded interactions with the protein in the SpAR-IR than in SpGR-IR complex. There is also a strong interaction in residue s2A7 of SpGR-IR which is not present in SpAR-IR. These differences in the protein-DNA interaction between the SpAR-IR and SpGR-IR complexes, that is two models of the same system, may represent two slightly different binding modes, as a consequence of different initial conformations used in the simulations.

On the other hand, our results show that both the Sp(AR/GR)-IR complexes exhibit stronger hydrogen-bond interactions than the GR-IR complex (compare residues s1G2 and s1G3, between Sp(AR/GR)-IR and GR-IR, residue s1T10 between SpAR-IR and GR-IR, and residue s2T6 and s2A7 between SpGR-IR and GR-IR). Furthermore, the AR-DR complex exhibits slightly stronger hydrogen-bond interactions than observed in the SpGR-DR but considerably stronger than observed in SpAR-DR. Interestingly, those interactions, present in AR-DR but not in SpAR-DR, are mostly formed with the HS1 and the spacer. Moreover, there are more water-mediated interactions in SpAR-IR than in SpGR-IR. Finally, the number of water-mediated hydrogen bond interactions in AR-DR is higher than in GR-IR.

## 4. Discussion

All the protein-DNA complexes modeled in this work, represent states in which the DNA is bound by the respective DBD. The interaction strengths within the complexes, as manifested by hydrogen bond interactions between protein and DNA, as well as between the protein subunits, and conformational flexibility, however, varies between the different systems.

Of all the protein-DNA systems, the AR-DR complex exhibits the strongest interactions between protein and the DNA via direct and water-mediated hydrogen bonds.

### 4.1. Protein–Protein Interactions

The complex which exhibits the strongest hydrogen bonds between the two protein monomers is AR-DR. In particular, the strong hydrogen-bonded interaction S580-S580, as suggested by the crystal structure (Shaffer et al., 2004), contributes to the stabilization of the dimerization interface. This interaction can also be regarded as facilitating the interaction of the neighboring R581 with D583. This is furthermore in agreement with the experimental suggestion that the strong dimer interface of AR-DR allows the AR-DBDs to bind to DNA in a head-to-head conformation (Shaffer et al., 2004; van Royen et al., 2012).

The mutations in the SPARKI systems, which transform an AR into the chimeric protein, are mainly located in one loop that constitutes the dimerization interface. The protein-protein interactions in all the SPARKI systems are weaker than in the AR-DR and comparable to (or even weaker than) those in the GR-IR system. This suggests that the dimerization interface of SPARKI is indeed GR-like, as would be expected based on its constituting sequence.

A significant conformational distortion can be seen in monomer A and the dimer interface of SpGR-DR, that is not observed in the SpGR-IR. In addition, the dimer interface of SpGR-DR has two hydrogen bonds fewer than the SpGR-IR. The SpGR-DR model, moreover, exhibits the largest Zn-Zn distances and the largest distance between the loops of the dimerization interface of all the models investigated in this work. These findings suggest that in the SpGR model, accommodation of the DR sequence, and interactions with the protein comparable to a IR sequence, can be achieved only at the expense of a distortion of the dimerization interface.

The deformation of monomer A and the dimerization interface observed in the SpGR-DR model is not observed in the SpAR-DR model, that is the complex that has been modeled from the crystal structure of the AR-DR. We attribute this difference to the different starting points for the simulations, AR-DR and GR-IR, respectively. In the SpAR models, the residues which have been *in silico* mutated (second zinc-binding motif) are located at the dimerization interface, whereas in the SpGR models these residues (first zinc-binding motif) are part of the DNA-binding interface. Furthermore, in the SpGR-DR model the DNA sequence has been changed from IR to DR *in silico*.

In the SpAR-DR model, the monomers of SpAR are tightly bound in the AR-like starting conformation. The modified dimerization interface leads to a weaker protein-protein interaction as manifested by the longer distance and fewer hydrogen bonds between the two subunits. The protein, on the other hand, does not “reach” the DNA as good as in the other models as can be seen by SpAR-DR showing the longest, though not by much, protein-DNA distances of all the complexes. Moreover, the number of hydrogen bonds between protein and DNA is smaller than in the wild-type AR-DR, in particular in HS1, pointing toward a loser complex in the chimeric model. This is in agreement, albeit does not fully explain the experimentally observed low affinity of SPARKI for DR elements (Schauwaers et al., 2007; Moehren et al., 2008; Sahu et al., 2014).

In the SpGR-DR model the dimerization interface is GR-like, that is weak to start with. In addition the protein is not properly oriented on the DR sequence. In the course of the simulation, the protein undergoes conformational changes in the dimerization interface, considerably weakening the protein-protein interactions. The distortion, weakened interactions in the dimerization interface, result in a reoriented monomer A and a deformed monomer B. That means that monomer B in SpGR does not manage to fully adjust onto the direct repeat on HS2 to form strong contacts. The observed conformational change in the Dim regions and the monomer A may be regarded as an attempt by the system to make favorable contacts in other parts of the complex. Indeed in the SpGR-DR model, more contacts, that is hydrogen-bonds between protein and DNA, are observed than in the SpAR-DR model. However, these contacts are with the HS1. Strong interactions with only one hexamer and a distorted protein-protein interface suggest a low affinity, or a rather unstable Sp(GR)-DR complex. The SpGR model is, by construction, a GR-like SPARKI. Also GR lacks affinity for DR sequences, possibly because no stable complexes can be formed between GR and DR. A deformed conformation in the dimerization interface of SpGR-DR may thus point toward a loss of stability in that wild-type GR-DR complex.

Analysis of the DNA parameters around T12 exhibits extreme values in the neighboring G11 (intra bp) as well as extreme inter base pair parameters in the GT step that are not present in the GA step of the direct repeat. The affected G11 has strong interactions with the protein and is therefore an important residue for binding. This interplay may explain why T12 is essential for specific DNA recognition by GR (Sahu et al., 2014) as has been shown by *in vivo* experiments.

The sequence and conformation in the HS2, moreover, affect the spacer region. In this region, a narrower major groove has been observed for the IR sequence than for the DR sequence. Such a DNA conformation, though not quite a kink in the DNA spacer, requires the protein to “follow” the DNA conformation so as to form favorable contacts. This is achieved by a lever arm that is more flexible in the IR-bound systems, i.e., GR and SPARKI (see Figure 3), and the two protein subunits being slightly further apart, as manifested by longer monomer-monomer distances in GR-IR compared to AR-DR, while the distances of the protein subunits to their respective half site on the DNA are similar. Among the complexes with an IR sequence, both SPARKI models, SpAR-IR and SpGR-IR, reveal stronger protein-DNA interactions, especially with the HS1, than the other wild-type complex, GR-IR, in agreement with experiments that show similar or higher affinity of SPARKI systems for the IR elements or classical response element, i.e., CREs (Schauwaers et al., 2007).

The higher affinity of the SpAR/GR complexes to the IR sequence, compared to that of GR-IR, can thus be explained by the chimeric systems having both properties, the AR-like ability to strongly interact with the DNA and the GR-like “softness”, that is weaker interactions, of the dimerization interface, that allows the protein to flexibly accommodate to the binding on the DNA. Qualitatively, the higher flexibility in the dimerization interface and lever arm region of the SPARKI-IR systems can be understood as entropically favorable. Indeed, the SPARKI models show a higher entropy than the wild-type complexes. Additionally, the stronger protein-DNA interactions can be understood as an increased enthalpic contribution. An increased binding affinity of SPARKI compared to GR can thus be attributed to favorable enthalpic and entropic contributions.

The AR-DR complex, in contrast, is more enthalpically stabilized by the contribution of both, protein-protein and protein-DNA hydrogen-bond interactions. In the DR-DNA the minor groove is ~1Å narrower at the GA step than at the corresponding GT step in an inverted repeat DNA. This narrower minor groove is associated with the phosphate groups of the DNA backbone being closer to each other, and thus providing a higher negative charge density. Electrostatic interactions of the positively charged Arg (and Lys at other positions) residues with the DNA is therefore strengthened, as manifested by the larger number of strong hydrogen bonds in the AR-DR system.

The protein-DNA complexes studied in this work are characteristic for a competition between the protein-protein interactions and protein-DNA interactions, that is, a stable dimerization interface vs. specific contacts to the DNA. A balance to the former or the latter thus decides about specificity, or at least preference, for direct or inverted repeat DNA, respectively.

## 5. Conclusion

Our simulations of the chimeric SPARKI protein, complexed to inverted and direct repeat sequences, reveal a higher affinity of this model protein for IR than for DR sequences. In fact, binding to a DR results in a loose complex, eventually even with a distorted protein conformation, a possible explanation for the experimentally observed weak affinity for such a sequence (Schauwaers et al., 2007; Moehren et al., 2008; Sahu et al., 2014).

Since AR, GR, and the SPARKI models can in principle all form the same contacts with specific residues of the DNA, IR or DR, the ability to accommodate the protein on the DNA is important for specificity. The required flexibility is observed in those systems with a “weaker” dimerization interface, that is GR and the GR-like SPARKI, which can thus be considered to have more entropy driven specificity. The interactions in the dimerization interface and protein-DNA interactions are balanced to allow proper accommodation of the protein on the DNA and formation of specific contacts, tuning the enthalpic contribution to specific complex formation. In this competition, the stability of the dimerization interface is important and to a large extend determines the preferred response element.

The starting point, that is the crystal structure used for model building, has, even after rather long simulation time, still an effect on the protein conformation in the complex. SPARKI models initiated from the structure of the GR-IR complex are not capable of forming strong interactions in the dimerization domain. In contrast, SPARKI models started from an AR-DR complex structure maintain a rather stable dimerization interface, despite the mutation of some residues in this domain to those of GR. Still, this interface is weaker than in the wild-type AR-DR complex,. Moreover, the chimeric SPARKI protein shows fewer interactions with DR than observed in AR-DR, rendering its specificity GR-like.

All together, this study reveals the importance of the dimerization domain on distinct specificity of AR and GR, bound to DR and IR response elements, respectively.

## Data Availability Statement

The datasets generated for this study will not be made publicly available. Datasets are available on request.

## Author Contributions

PI and MB designed the research. MB performed the research. MB and SV analyzed the data. MB and PI wrote the manuscript.

### Conflict of Interest

The authors declare that the research was conducted in the absence of any commercial or financial relationships that could be construed as a potential conflict of interest.
