# Supplementary material for: Molecular Dynamics Simulations of a Chimeric Androgen Receptor Protein (SPARKI) Confirm the Importance of the Dimerization Domain on DNA Binding Specificity
Source: Front Mol Biosci. 2020 Jan 31;7:4. doi: 10.3389/fmolb.2020.00004 (PMC7005049; doi:10.3389/fmolb.2020.00004)
Supplement: Supplementary file 1 [file Data_Sheet_1.pdf]

Supplementary Information for: Molecular Dynamics Simulations of a Chimeric Androgen Receptor Protein (SPARKI) confirm the Importance of the Dimerization Domain on DNA Binding Specificity

Mahdi Bagherpoor-Helabad*, Senta Volkenandt, and Petra Imhof*, §

Department of Physics
Freie Universität Berlin
Arnimallee 14
14195 Berlin
Germany

§ Present address: Department of Chemistry, Bioscience, and Environmental Engineering, University of Stavanger, P.O. Box 8600 Forus, N-4021 Stavanger, Norway

* Corresponding author

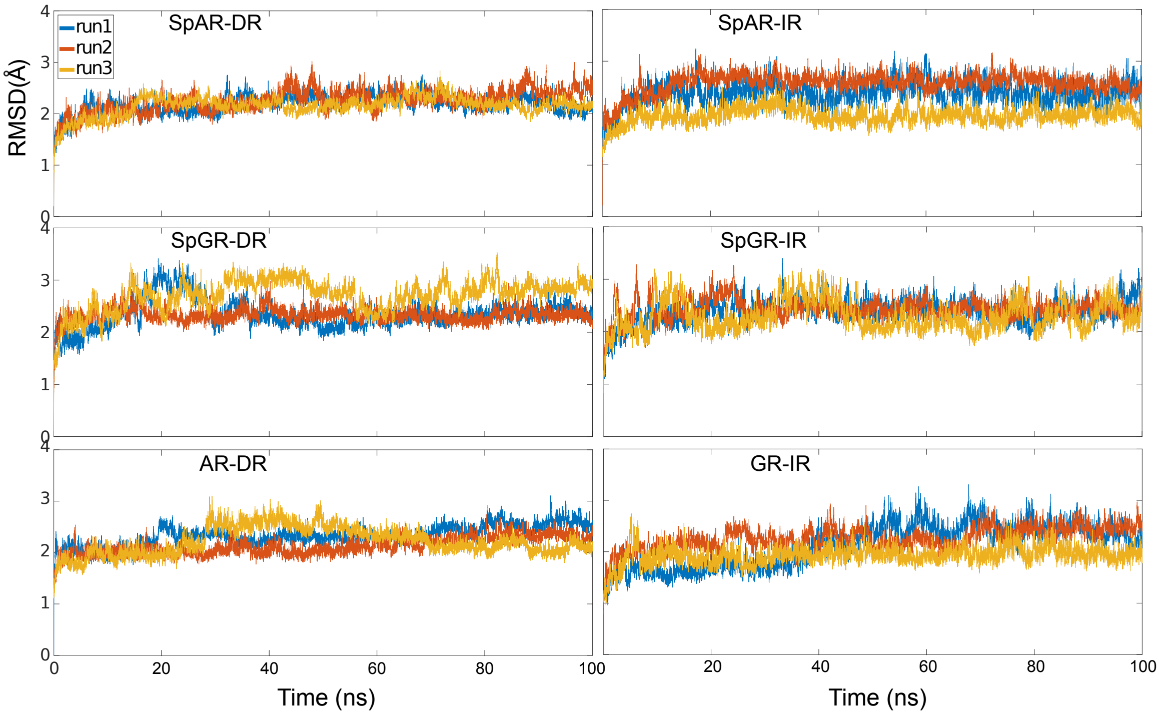

Figure S1: Root mean square displacement of three 100 ns replicas MD simulations, for each system. For SpAR-DR, SpGR-DR, SpGR-IR, and AR-DR, run1 is selected for carrying out the longer simulations and for SpAR-IR and GR-IR, run3 is selected for carrying out the longer simulations.

The DNA geometries of the SPARKI systems exhibit different values of parameters in the three time intervals: the first (W1), middle (W2), and last 100 ns (W3) windows of the trajectories. The chimeric SPARKI systems are based on AR-DR and GR-IR systems and are thus anticipated to show a longer relaxation time in the simulations. However, for IR complexes, the results of intervals W2 and W3 show almost the same values in DNA parameters (Figure 6 and Figures S3, S4(b,d)) whereas for DRs, the values in the W3 differ from those observed in the earlier W1 and W2 intervals (Figure 6 and Figures S3, S4(a,c)). An exception is the SpGR-IR model for which we observe in the W3 interval bending parameters different from those in intervals W1 and W2, see SI Figure S4(d).

Comparison of the DNA parameters of SpGR-DR in the W3 interval with those in the W1 interval shows a considerable change in DNA geometry in the course of the simulation. As our results indicate, the DNA of both SPARKI-DR complexes also has a geometry that is different from the DNA in the AR-DR system. However, these differences are considerably larger between SpGR-DR and AR-DR than between SpAR-DR and AR-DR.

For SpAR-DR both groove widths in the W3 interval show only small differences with respect to the W1 interval, although the DNA considerably loses its bending at the end of simulation, i.e. in the W3 interval (see Figures S2, S3, and S4(a)). In the SpGR-DR complex, the DNA shape

Table S1: Average distance between different domain/subdomains (as characterized by the distances between the respective centers of mass) of the protein-DNA complexes.

Distance (Å)	**AR-DR**		**SpAR-DR**		**SpAR-IR**	
	mean	std	mean	std	mean	std
Monomer A - HS1	16.89	0.19	17.04	0.21	16.68	0.20
Monomer B - HS1	16.62	0.21	17.08	0.19	16.75	0.18
Monomer A - Monomer B	24.37	0.31	24.08	0.24	23.68	0.25
Dimer interface	9.70	0.26	9.97	0.20	9.69	0.22
ZN_A-ZN_B (Dim)	9.06	0.30	9.35	0.27	9.19	0.30
Distance (Å)	**GR-IR**		**SpGR-DR**		**SpGR-IR**	
	mean	std	mean	std	mean	std
Monomer A - HS1	16.42	0.19	16.78	0.23	16.68	0.20
Monomer B - HS1	16.27	0.17	16.97	0.26	16.75	0.18
Monomer A - Monomer B	25.08	0.20	24.45	0.39	24.04	0.32
Dimer interface	9.88	0.18	10.47	0.33	9.87	0.43
ZN_A-ZN_B (Dim)	9.08	0.24	10.20	0.50	9.33	0.36

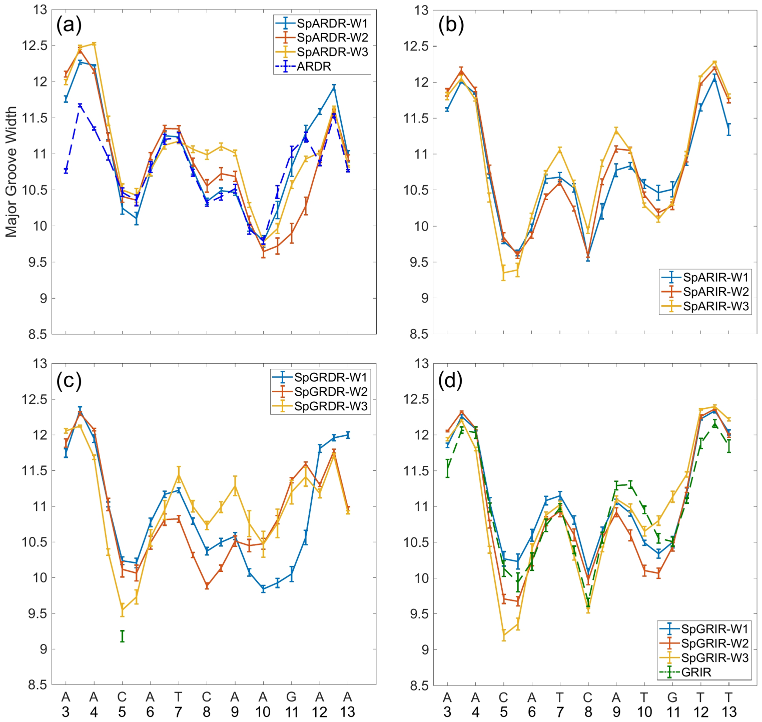

Figure S2: Comparison of the DNA major groove for (a) SpAR-DR and AR-DR (dashed line colored in blue) (b) SpAR-IR (c) SpGR-DR (d) SpGR-IR and GR-IR (dashed line colored in green). For SPARKI systems, the lines colored in light blue, red, and orange correspond to the major groove analysis for the first 100 ns (W1), middle 100 ns (W2), and last 100 ns (W3) intervals of 900 ns MD simulations, respectively. For AR-DR and GR-IR, the results are calculated for the last 100 ns of the 500 ns MD simulations.

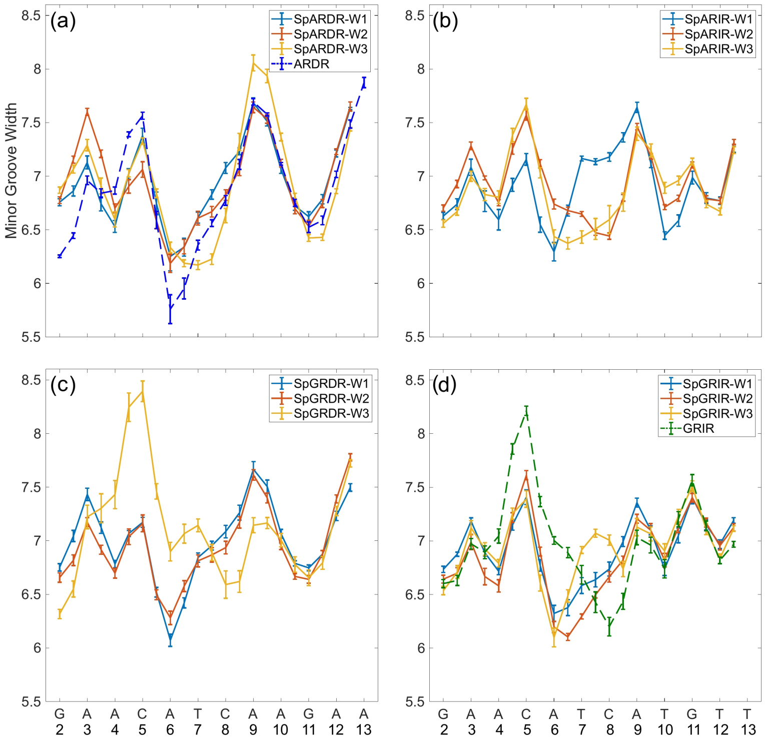

Figure S3: Comparison of the DNA minor groove for (a) SpAR-DR and AR-DR (dashed line colored in blue) (b) SpAR-IR (c) SpGR-DR (d) SpGR-IR and GR-IR (dashed line colored in green). For the SPARKI systems, the lines colored in light blue, red, and orange correspond to the minor groove analysis for the first 100 ns (W1), middle 100 ns (W2), and last 100 ns (W3) intervals of 900 ns MD simulations, respectively. For AR-DR and GR-IR the results are calculated for last 100 ns of 500 ns MD simulations.

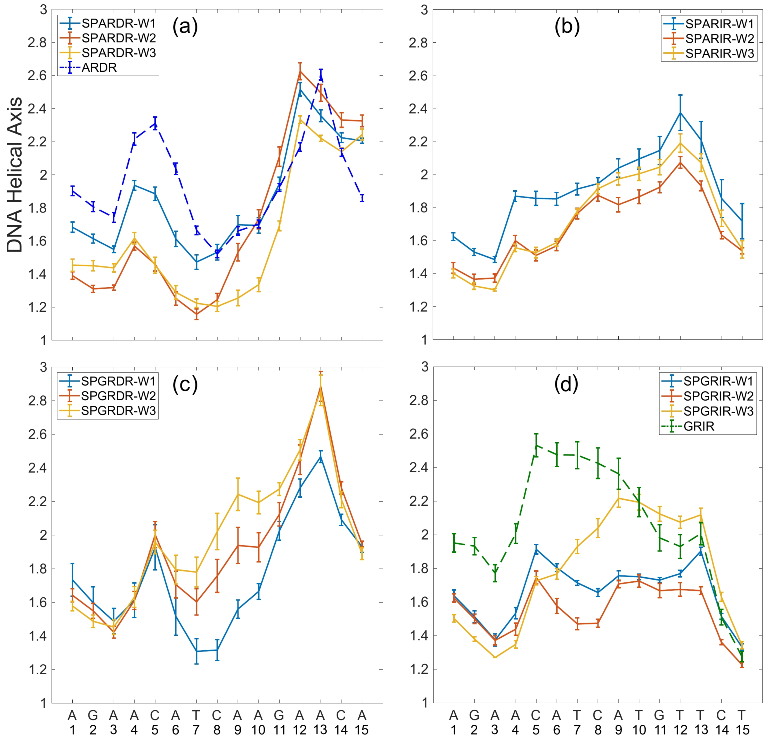

Figure S4: Comparison of the DNA helical axis bending for (a) SpAR-DR and AR-DR (dashed line colored in blue) (b) SpAR-IR (c) SpGR-DR (d) SpGR-IR and GR-IR (dashed line colored in green). For SPARKI systems, the lines colored in light blue, red, and orange corresponds to DNA helical axis bending analysis for the first 100 ns (W1), middle 100 ns (W2), and last 100 ns (W3) intervals of 900 ns MD simulations, respectively. For AR-DR and GR-IR the results are calculated for last 100 ns of 500 ns MD simulations.

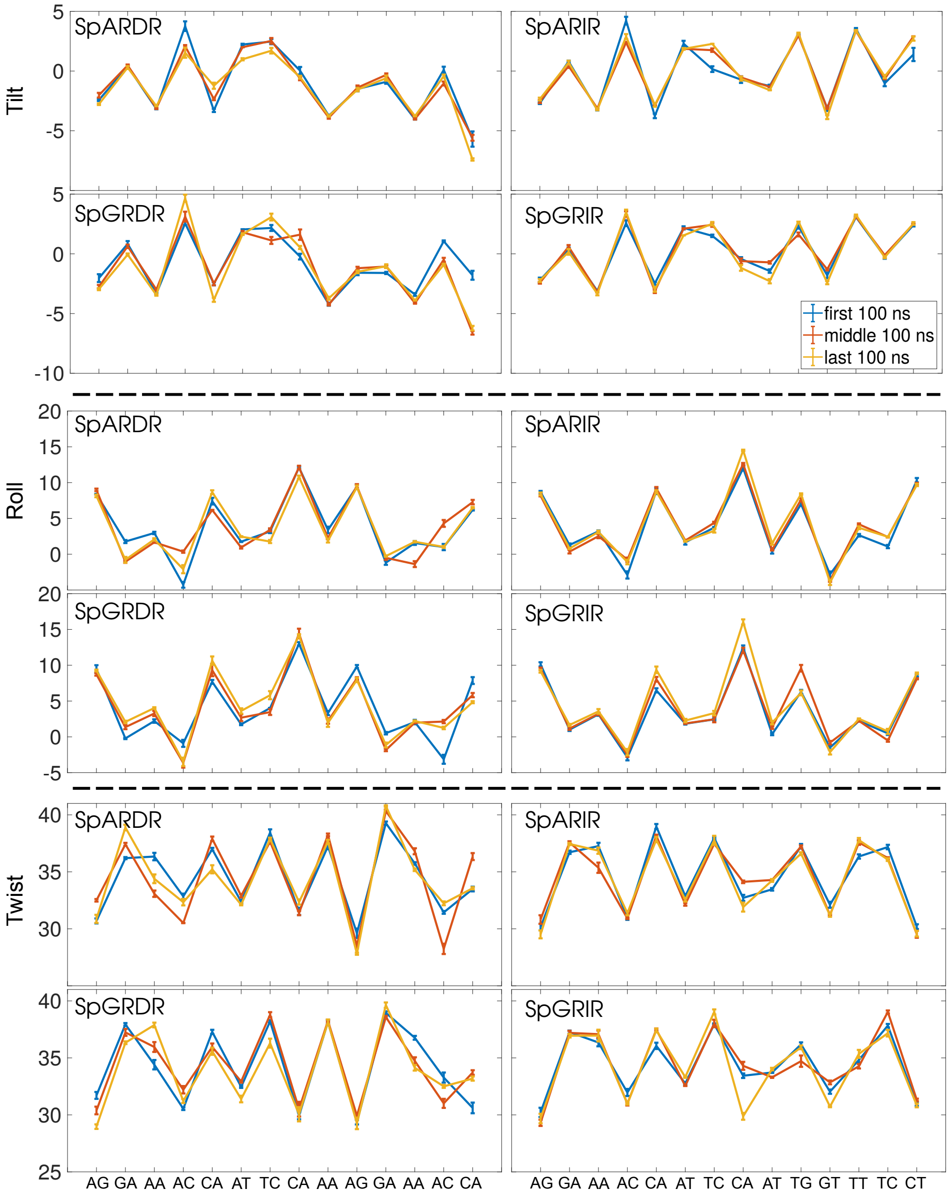

Figure S5: DNA rotational inter base pair parameters for SPARKI models.

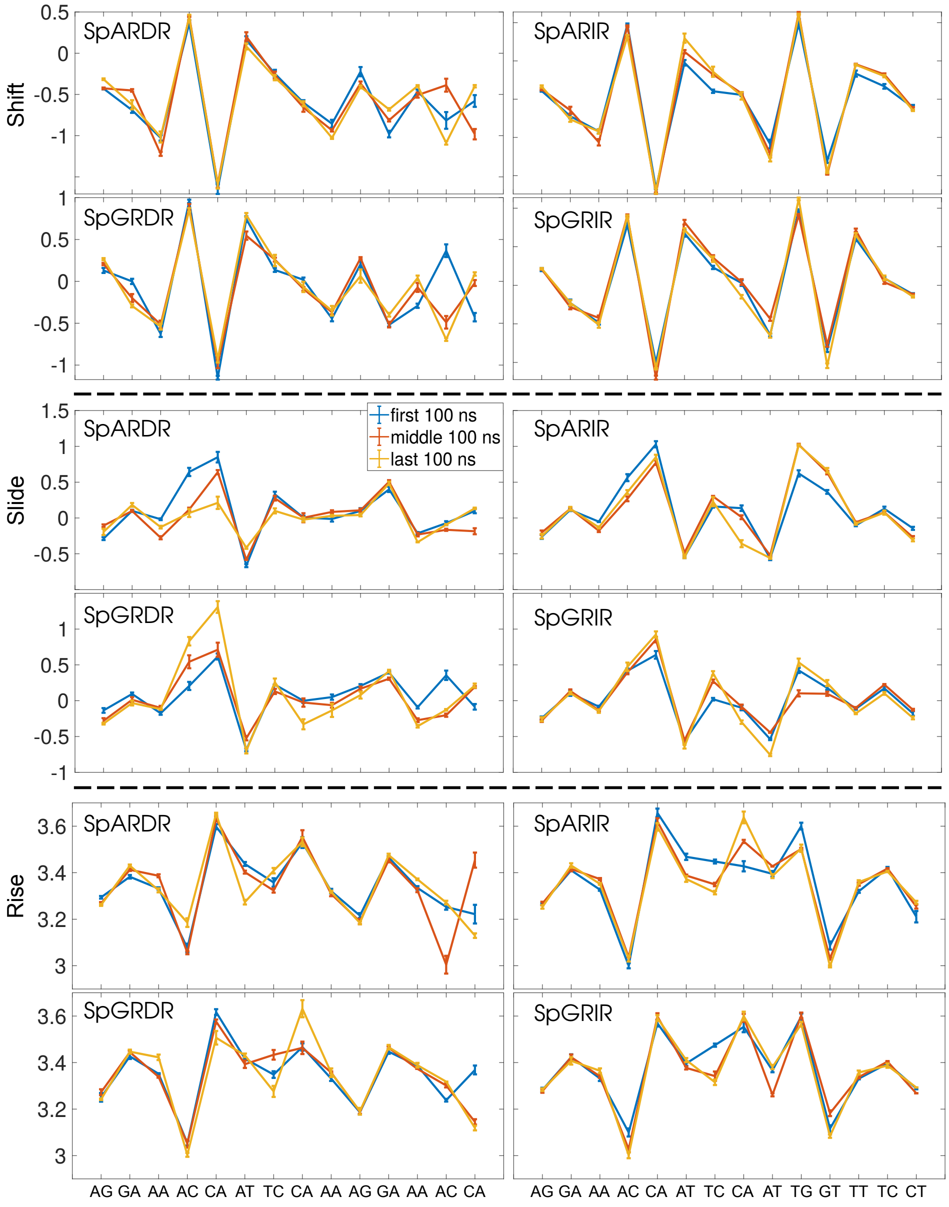

Figure S6: DNA translational inter base pair parameters for SPARKI models.

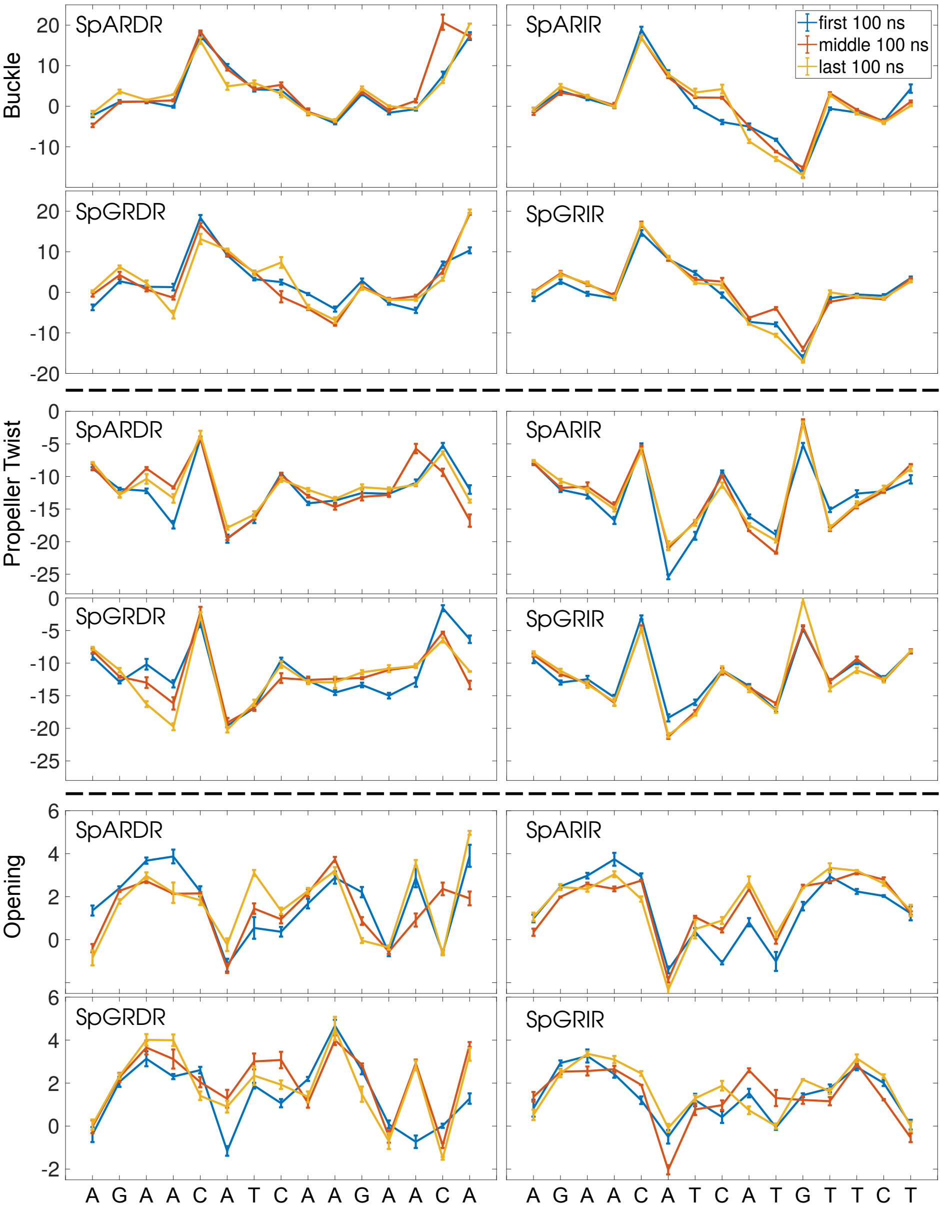

Figure S7: DNA rotational intra base pair parameter fors SPARKI DNA.

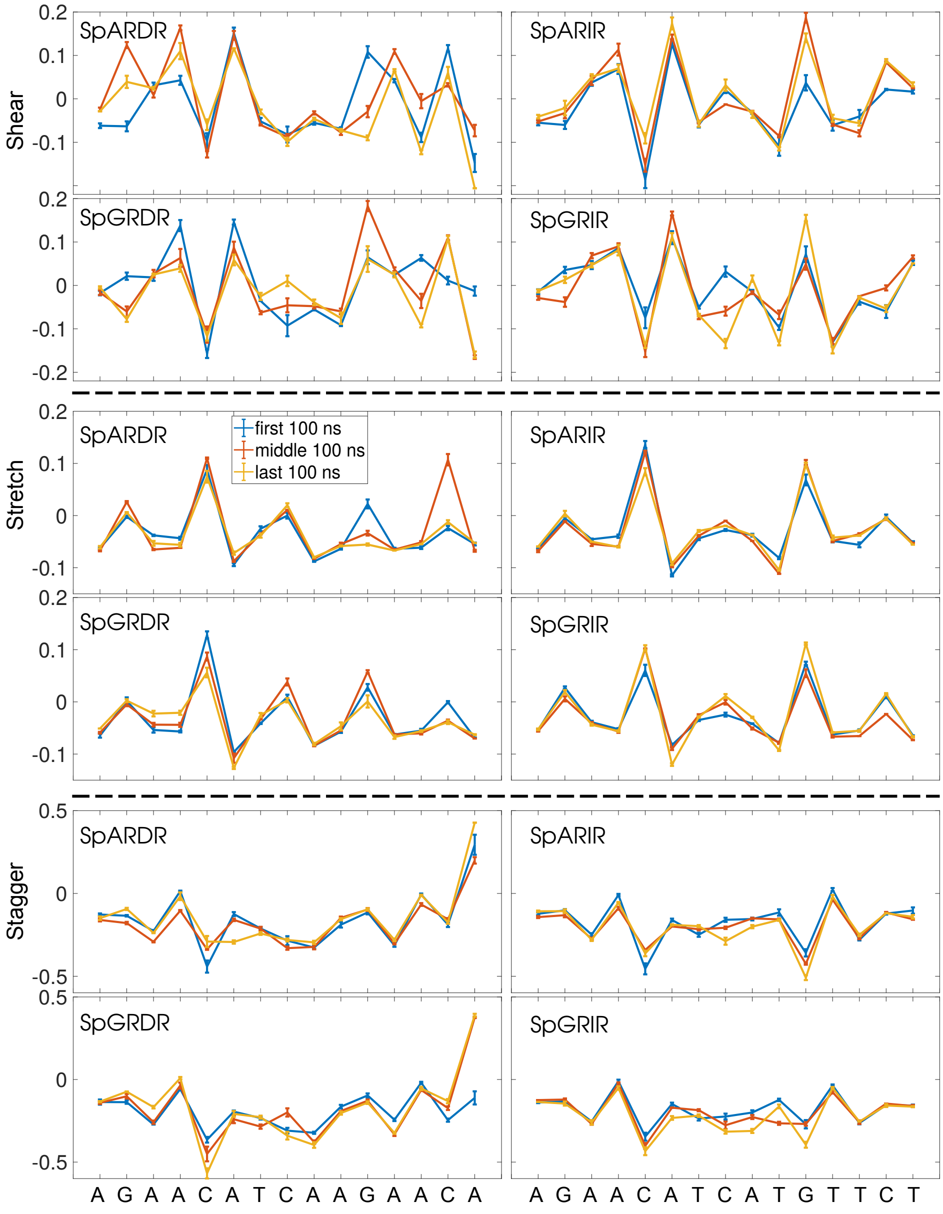

Figure S8: DNA translational intra base pair parameters for SPARKI models.

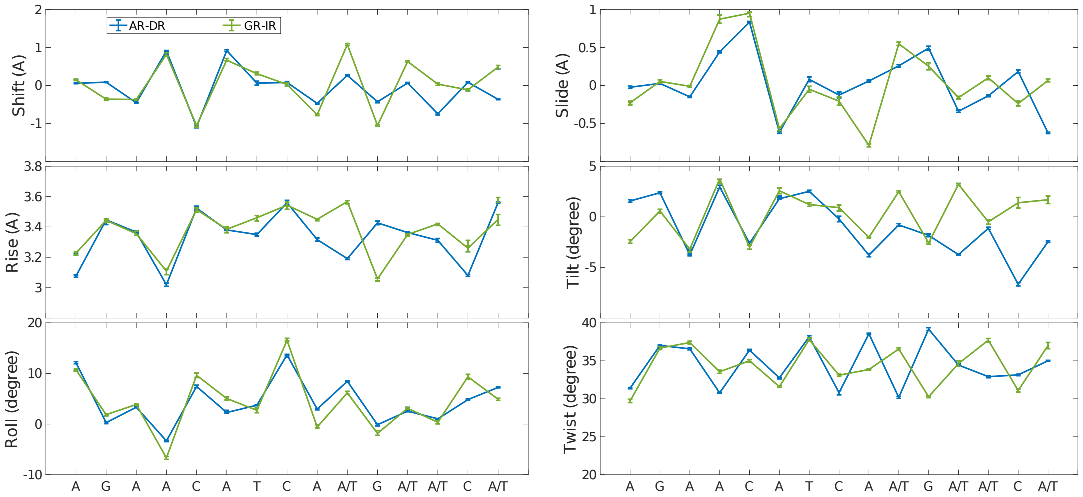

Figure S9: DNA translational and rotational inter base pair parameter fors AR-DR and GR-IR models.

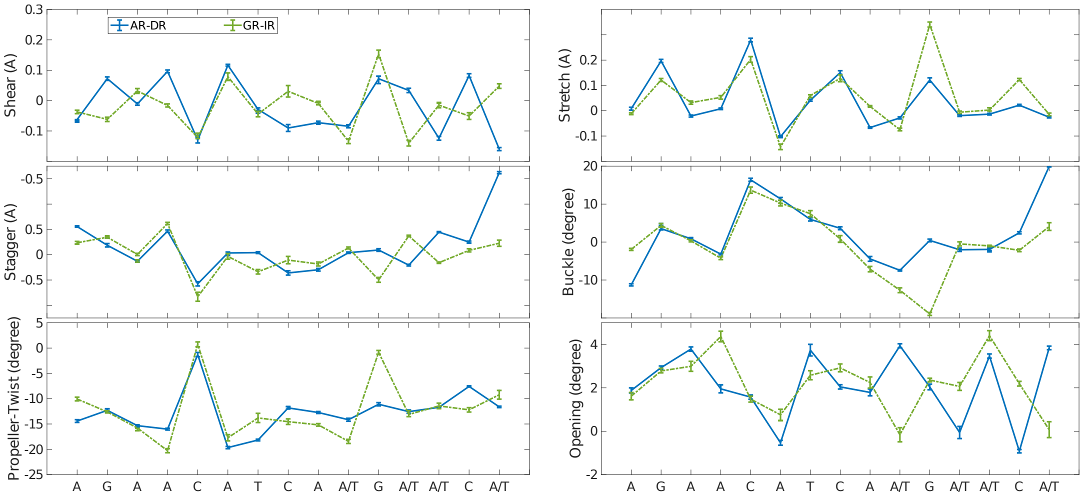

Figure S10: DNA translational and rotational intra base pair parameters for AR-DR and GR-IR models.

is significantly changed from the W1 and W2 intervals to the W3 interval. As our results show, the DNA of both Sp(AR/GR)-DR complexes, also show different conformations with respect to the DNA of the AR-DR system. For SpAR-DR, the most significant change happens in the DNA bending parameters, as can be seen from the comparison with AR-DR, Figure S4(a).

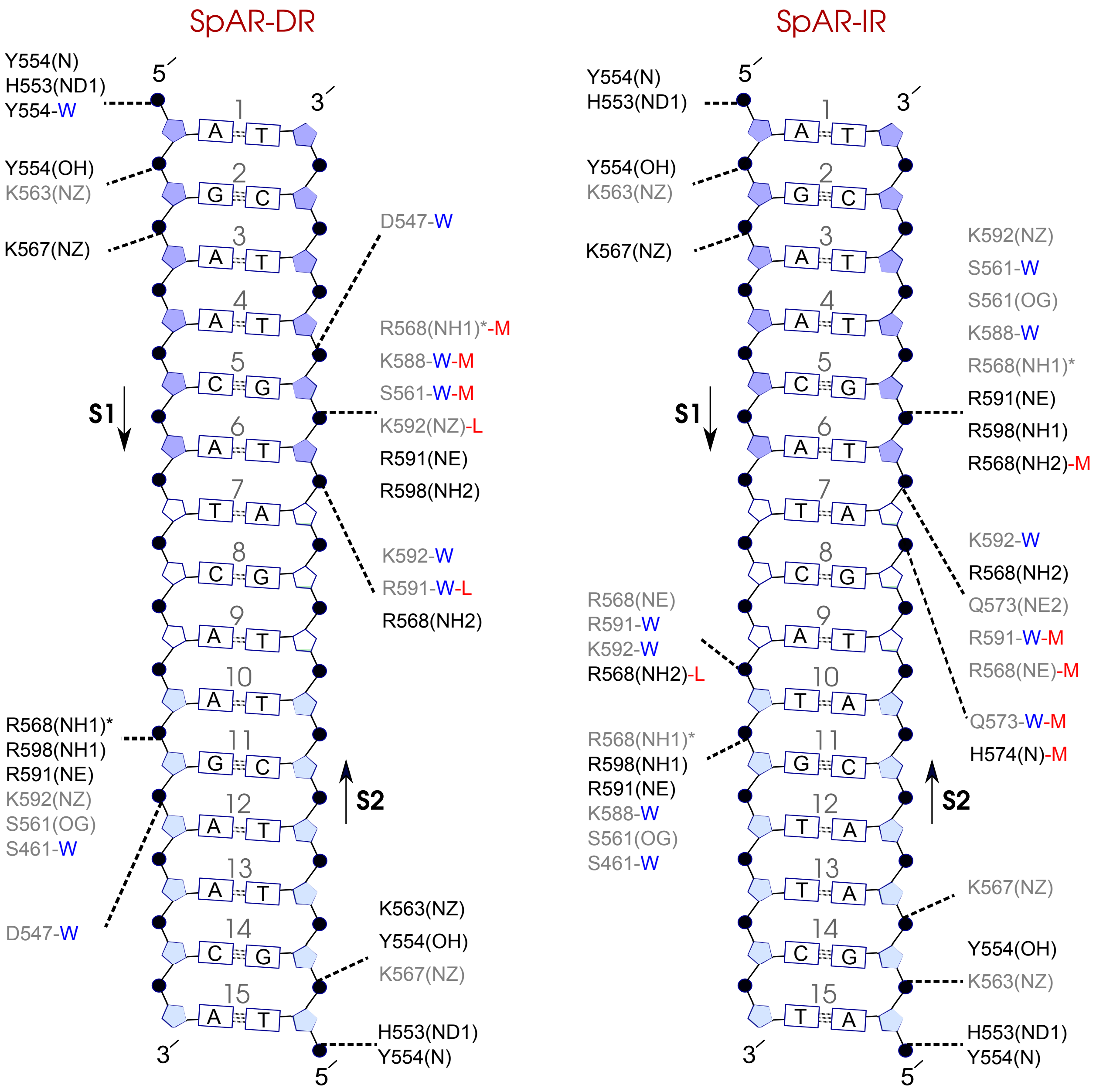

Figure S11: Diagram of protein-DNA hydrogen-bond interactions for (*left*) SpAR-DR and (*right*) SpAR-IR. The nucleotides of the 15 bps core DNA sequence are numbered from HS1 (numbers: 1 to 6) to HS2 (numbers: 10 to 15). The spacer region is highlighted with non-colored boxes around the numbers of the bases (numbers: 7-9). The hydrogen bonds are categorized based on their occupancy, 50-75% (gray), and 75-100% (black). The water mediated hydrogen bonds are labeled with a blue letter "W". The residues shown with star sign form base-specific hydrogen-bond interactions while the other residues form interactions with the backbone of DNA. The letters "M" and "L" colored in red show the hydrogen-bond interactions only seen in "W2" (middle 100 ns) and "W3" (last 100 ns) time interval of trajectories, respectively. All other interactions are observed in both "W2" and "W3" intervals.

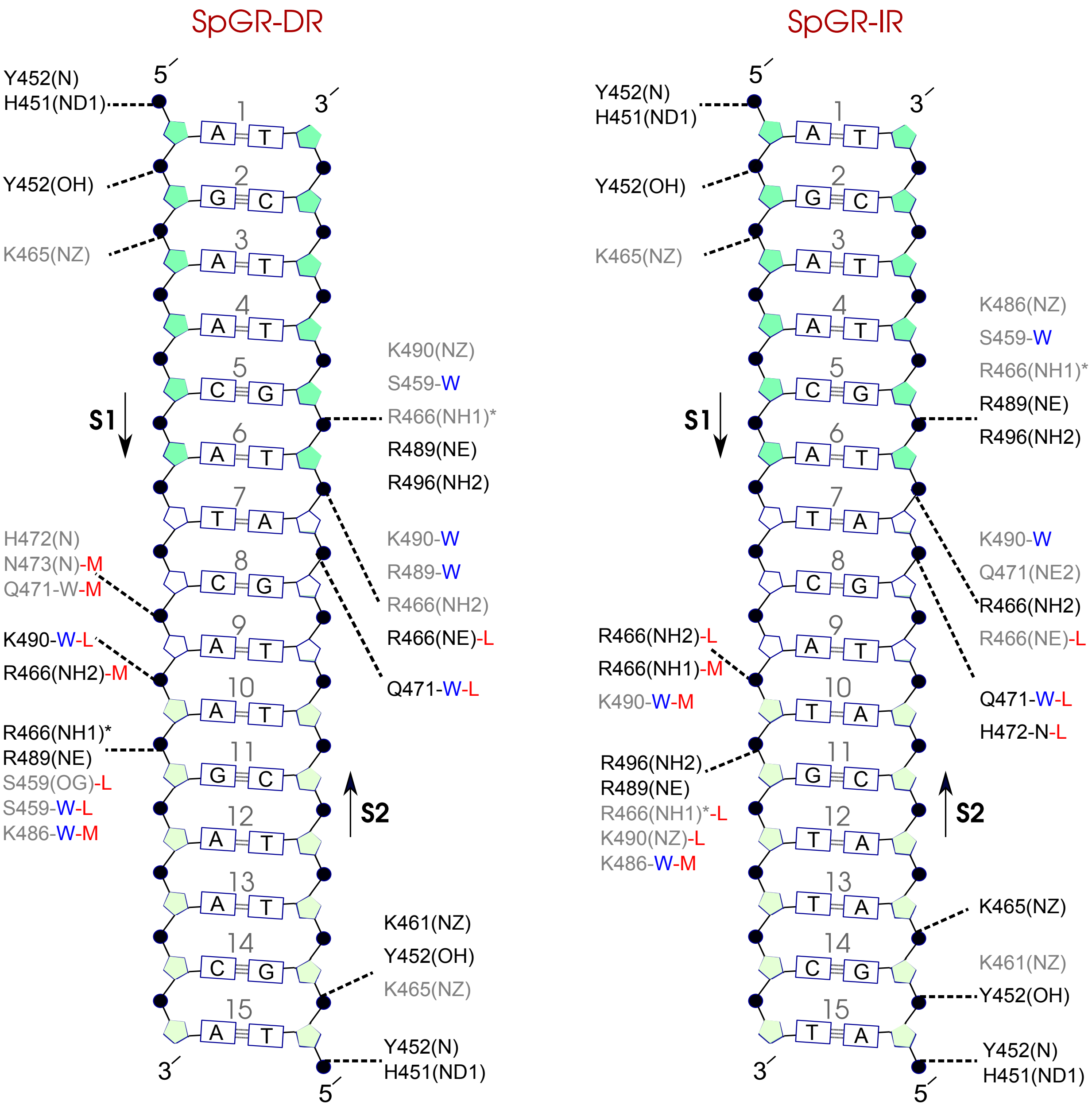

Figure S12: Diagram of protein-DNA hydrogen-bond interactions for (*left*) SpGR-DR and (*right*) SpGR-IR. The nucleotides of the 15 bps core DNA sequence are numbered from HS1 (numbers: 1 to 6) to HS2 (numbers: 10 to 15). The spacer region is highlighted with non-colored boxes around the numbers of the bases (numbers: 7-9). The hydrogen bonds are categorized based on their occupancy, 50-75% (gray), and 75-100% (black). The water mediated hydrogen bonds are shown with a blue letter "W". The residues shown with star sign form base-specific hydrogen-bond interactions while the other residues make interactions with the backbone of DNA. The letters "M" and "L" colored in red show the hydrogen-bond interactions only seen in "W2" (middle 100 ns) and "W3" (last 100 ns) time interval of trajectories, respectively. All other interactions are observed in both "W2" and "W3" intervals.

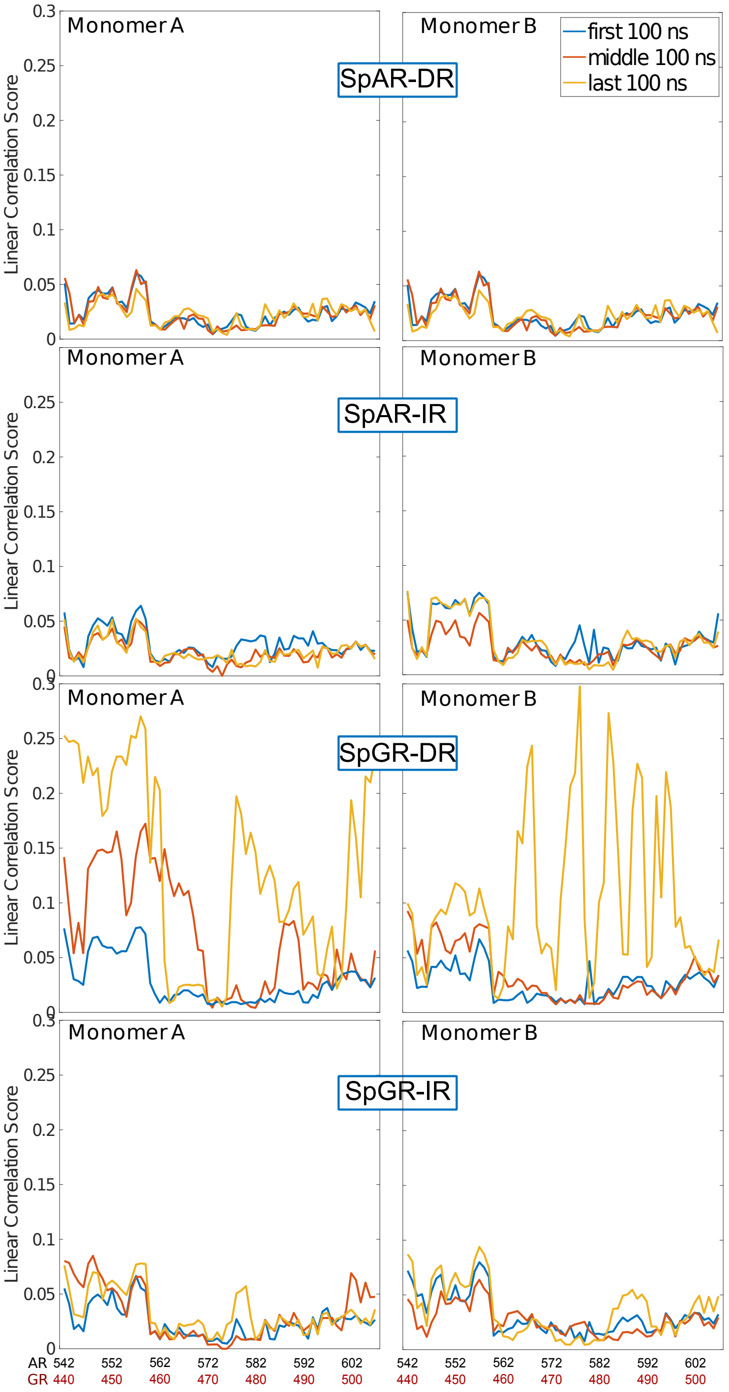

Figure S13: Correlation score per residue, computed for intra-domain correlations with $r_{ki} \geq 0.4$ for SPARKI system, calculated for first 100 ns ("W1"), middle 100 ns ("W2"), and last 100 ns ("W3") time intervals of 900 ns SPARKI's MD trajectories.
